# Medication adherence scales in non-communicable diseases: A scoping review of design gaps, constructs and validation processes

**DOI:** 10.1371/journal.pone.0321423

**Published:** 2025-05-14

**Authors:** Maria Jose, Priyanka Rajmohan, T. S. Sulfath, Ravi Prasad Varma, Manoj Mohan, Nisha K. Jose, Jerin Jose Cherian, Mohan Lal Bairwa, Tulika Goswamy, Aditi Apte, Praveenlal Kuttichira, Joe Thomas

**Affiliations:** 1 Department of Pharmacology, Jubilee Mission Medical College & Research Institute, Thrissur, India; 2 Department of Community Medicine, Jubilee Mission Medical College & Research Institute, Thrissur, India; 3 Achutha Menon Centre for Health Science Studies, Sree Chitra Thirunal Institute for Medical Sciences and Technology, Trivandrum, India; 4 Department of Obstetrics and Gynecology, Aster Hospital, Doha, Qatar; 5 Indian Council of Medical Research, New Delhi, India; 6 Clinical Studies and Trials Unit, Division of Development Research, Indian Council of Medical Research, New Delhi, India; 7 Department of Global Public Health, Karolinska Institute, Stockholm, Sweden; 8 Centre for Community Medicine, AIIMS, New Delhi, India,; 9 Department of Community Medicine, Assam Medical College, Dibrugarh, India; 10 KEM Hospital Research Center, Pune, India; 11 Department of Psychiatry, Jubilee Mission Medical College & Research Institute, Thrissur, India; Xiamen University—Malaysia Campus: Xiamen University—Malaysia, MALAYSIA

## Abstract

**Introduction:**

NCDs arise from complex interactions of modifiable factors such as unhealthy lifestyles, poor diet, and psychosocial challenges, along with non-modifiable factors like age and genetics. Notably, medication non-adherence is a widespread and growing concern, significantly contributing to disease progression and poor outcomes globally.

**Objective:**

This scoping review aims to synthesize evidence on medication adherence scales used for selected non communicable diseases. It examines their development methods, psychometric properties, and assessed domains, while identifying gaps or limitations in their design and application.

**Materials and methods:**

The Joanna Briggs Institute methodological framework guided this scoping review and the protocol was registered prospectively to ensure methodological transparency and rigor. Electronic databases, the reference list of included articles, and grey literature were searched. Studies published in English from January 1950 to June 2024 were included. Two reviewers independently screened all articles, and a third reviewer settled any conflicts between the reviewers. Critical appraisal of the screened-in articles was done using JBI critical appraisal scales. The data was compiled into tables and a narrative summary that is consistent with the review’s goal.

**Results:**

Our study included 140 articles, identifying 57 medication adherence scales. These scales, developed using qualitative methods (10.8%), literature review (32.4%), and mixed methods (45.9%), primarily focus on behavior, often neglecting cost-related non-adherence, self-efficacy, and systemic barriers. Psychometric findings varied widely, reflecting heterogeneity in study designs and scale development approaches. Many scales lack validation in diverse settings, underscoring the need for comprehensive, context-sensitive tools.

**Conclusion:**

This scoping review highlights gaps in existing medication adherence scales for NCDs, particularly their limited consideration of socioeconomic and cultural factors and incomplete adherence assessment. Future research should focus on developing more holistic, contextually relevant adherence scales that integrate these dimensions. Strengthening adherence measurement methodologies can enhance patient-centered care, inform policy interventions, and improve health outcomes.

## Introduction

### Background

‘Medication adherence’ is defined as the extent to which a person’s medication-taking behavior corresponds with agreed recommendations from a healthcare provider [1]. Adherence to medication is a crucial aspect of patient care and is indispensable for achieving clinical goals. The World Health Organization’s report on medication adherence states that “increasing the effectiveness of adherence interventions may have a far greater impact on the health of the population than any improvement in specific medical treatment” [1]. However, adherence rates remain suboptimal worldwide. In developed countries, only 50% of patients treated for chronic diseases adhere to prescribed treatments, with adherence rates being even lower in developing countries [2].

Non-communicable diseases (NCDs) contribute to a significant proportion of morbidity and mortality, with inadequate control of these conditions often linked to poor medication adherence. Socioeconomic constraints, language barriers, multimorbidity, mental health challenges, limited healthcare access, and lifestyle factors all influence adherence [3,4]. Patients with limited financial resources may be forced to prioritize daily expenses over purchasing medications, leading to treatment discontinuation [5].

Additionally, language barriers hinder effective communication between patients and healthcare providers, increasing the risk of misinterpretation of prescription instructions and improper medication use [6]. Patients with multimorbidity face greater medication burden, often requiring complex dosing regimens that heighten the risk of non-adherence due to polypharmacy and treatment fatigue. Studies have shown that as the number of prescribed medications increases, adherence tends to decline, particularly among elderly patients managing multiple chronic conditions [7].

NCDs contribute to around 38 million (68%) of all deaths globally and to about 5.87 million (60%) of all deaths in India [2]. Medication non-adherence rates range from 18.7% to 74% [8–11], with 30% of medicine-related hospital admissions attributed to non-adherence [12,13]. Given the dynamic nature of patient behaviors, accurately assessing adherence remains a challenge for healthcare professionals [14,15].

Non-adherence is multifactorial, influenced by patient beliefs, therapy-related barriers, asymptomatic conditions, and social determinants. Stigma, concerns about treatment efficacy, financial difficulties, and medication access further exacerbate the issue. A study conducted in Bangalore by Thomas D et al. [12] highlighted these barriers, reporting that 39.14% of patients were non-adherent due to specific beliefs about their treatment, 78.62% cited financial difficulties in affording medication, and 54.93% faced challenges in obtaining refills.

Measuring adherence is critical for understanding these challenges and designing effective interventions. While objective adherence measures (e.g., pill counts, electronic monitoring, biochemical tests) provide accuracy, they are resource-intensive [16–18]. Subjective measures, such as self-reports and healthcare assessments, are cost-effective, non-intrusive, and capture patient concerns, though they may be influenced by biases [19]. Selecting the appropriate adherence scale requires evaluating its development process, psychometric properties, and contextual applicability.

This scoping review aims to synthesize evidence on medication adherence scales used for major NCDs, specifically Type 2 Diabetes Mellitus (T2DM), Hypertension (HTN), Coronary Artery Disease (CAD), and Bronchial Asthma (BA)/Chronic Obstructive Pulmonary Disease (COPD). These conditions were chosen based on their high global prevalence [20], significant burden of nonadherence [10] and the distinct adherence challenges they pose—ranging from asymptomatic progression (HTN) to symptom-driven adherence patterns (BA/COPD) [21,22]. Additionally, these diseases often require long-term pharmacotherapy, making adherence measurement crucial for optimizing patient outcomes [23].

The global burden of NCDs, particularly in resource-constrained settings, emphasizes the need for culturally and contextually relevant tools. A detailed analysis of existing scales is necessary to identify their strengths, limitations, and gaps, enabling the development of robust, evidence-based tools that support tailored interventions and improve health outcomes while reducing healthcare costs. This would be done by considering their psychometric characteristics, including delineation of quality benchmarks such as sensitivity, specificity, convergent validity, and reliability metrics, along with the development process and the context of testing these scales.

By evaluating their strengths and limitations, this review aims to inform the selection and adaptation of adherence measurement tools, ultimately guiding the development of evidence-based interventions to improve adherence and health outcomes.

## Materials and methods

We prepared a study protocol and predefined the data sources, search strategy, study eligibility criteria, data extraction and criteria for quality assessment of the studies. This review was conducted following the Joanna Briggs Institute (JBI) methodology [14,24]. The reporting of the scoping review followed the Preferred Reporting Items for Systematic Reviews and Meta-Analyses extension for Scoping Reviews (PRISMA-ScR) (S3 Text) [25] and the protocol (S4 Text) was registered with Open Science Framework (OSF) (https://doi.org/10.17605/OSF.IO/VNMGH).

### Concept

The review considered the studies on the development and validation of all globally available medication adherence scales in selected NCDs (T2DM, HTN, COPD/ BA, CAD) to synthesize evidence using systematic search strategies, quality assessment, and data charting.

### Context

This scoping review considered medication adherence scales developed, validated and evaluated in the context of selected NCDs (T2DM, HTN, COPD/ BA, CAD) in community and hospital settings.

### Eligibility criteria/Inclusion criteria

English-language studies on the development or validation of adherence scales for adults with T2DM, HTN, COPD/BA, or CAD, published from January 1950 to June 2024, were included. Studies from 1950 onwards were included to ensure a comprehensive review of medication adherence assessment. While standardized adherence scales were developed later, earlier literature contributed to the conceptual understanding of adherence, influencing the design and validation of later tools.

The quality assessment of the included studies was conducted using the JBI critical appraisal criteria [14] (Table B, S1 Appendix) to ensure methodological rigor. As no predefined scoring system exists, we developed one based on expert judgment and established methodological frameworks (Table C, S1 Appendix). Although critical appraisal is optional in scoping reviews, we incorporated it to strengthen the synthesis rather than as an exclusion criterion [26]. All included studies were deemed to be of high quality.

For cohort studies, the parameters were (a) eligibility criteria (defined or not), (b) source of cohort (specified or not), (c) methods of selection and (d) methods of follow up. The maximum score possible was 4 and the minimum was 0. The parameters for quality criteria for case–control studies were (a) eligibility criteria (defined or not), (b) sources (methods of case ascertainment and control selection) and (c) rationale for the choice of cases and controls. The quality criteria for cross-sectional studies were (a) eligibility criteria, (b) sources and (c) methods of selection. The maximum score for case–control and cross-sectional study was 3 and the minimum was 0.

Studies that were not in English, focused on non-adult populations, addressed conditions outside the selected NCDs (T2DM, HTN, COPD/BA, or CAD) or did not involve the development or validation of adherence scales were excluded.

### Data sources

This scoping review considered quantitative studies, mixed methods studies, and systematic and scoping reviews. To ensure that no validated scales were overlooked, we initially considered SRs to identify any potentially unique scales discussed within them. Quantitative designs include any experimental study designs (e.g., randomized controlled trials, non-randomized controlled trials, or other quasi-experimental studies, including before and after studies), and observational designs (e.g., descriptive, cohort, and cross-sectional studies). Mixed methods include quantitative and qualitative designs used to validate medication adherence scales.

### Search strategy

A detailed search of MEDLINE (PubMed), Embase and Embase Classic, Scopus, Cochrane CENTRAL Register of Clinical Trials, and APA PsycINFO was conducted (Table A in S1 Appendix). The search for unpublished studies was carried out in GreyNet, OpenGrey, and Shodhganga. The reference list of included articles was also searched.

### Study selection/Screening

Development, validation and translational studies of medication adherence scales in the context of select NCDs (T2DM, HTN, COPD/ BA, CAD), published in peer-reviewed journals were included in this review. Two independent reviewers (STS and PR) systematically searched the literature using the prespecified strategy and scrutinized the titles and abstracts against the inclusion criteria for the scoping review. Full text of screened-in studies were obtained. Any disagreement between the two reviewers was confirmed by consulting a third reviewer.(JT) We eliminated duplicates using the Zotero Software version 12 (Thomson Reuters, New York). Critical appraisal of the screened-in articles was done using JBI critical appraisal scales.(STS and MJ)

### Data charting

The ‘descriptive-analytical’ method within the narrative tradition was utilized in data charting following the scoping review methodology established by JBI methodology for scoping reviews [24]. Two reviewers independently screened each study and independently mapped the studies (STS and PR) using the data extraction form, and any discrepancy between the reviewers was resolved by a third reviewer.(JT) The following data were extracted under different categories (S2 Dataset): 1)Study characteristics: author, year of publication, country, type of study, study setting, and sample size (Table 1). 2) Details of participants and scales: population, disease, Self-report scale, cut-off score, medication, age, gender, and percentage of non-adherence. 3) Psychometric properties of development and translational studies (Table 2 and Table E in S1 Appendix). 4) Medication adherence assessment scale comparison from development and translational validation studies: scale developed based on, number of questions, time to complete, and how scale administered (Table 3).

**Table 1:** Study characteristics.

Sl.No.	Self- report Scale	Author, Year	Country, Income country level	Single/ Multicentric	Development/ Translational validation/ MA assessment & validation	Type of study^a^	Sample size	Disease evaluated	% of non-adherence
1	MMAS 8	Sakthong P et al., 2009 [126]	Thailand, UMIC	Single	Validation study	Hospital based	303	T2DM	45.90
		Al Khazaz H et al., 2010 [51]	Malaysia, UMIC	Single	Validation study	Hospital based	223	T2DM	38.20
		Savoldelli V K et al., 2012 [168]	France, HIC	Single	Validation study	Hospital based	199	Hypertension	17.60
		Asilar R H et al., 2013 [78]	Turkey, LMIC	Multicentric	Validation study	Community based	196	Hypertension	–
		Lee W Y et al., 2013 [103]	Korea, HIC	Single	Validation study	Hospital based	321	T2DM	–
		Shin D S et al., 2013 [132]	South Korea, HIC	Multicentric	Validation study	Community based	92	Hypertension	33.70
		Deoliveira-Filho A D et al., 2014 [121]	Brazil, UMIC	Multicentric	Validation study	Community based	937	Hypertension	46.00
		Kim J H et al., 2014 [90]	South Korea, HIC	Multicentric	Validation study	Hospital based	373	Hypertension	–
		Arnet I et al., 2015 [60]	Switzerland, HIC	Single	Validation study	Hospital based	70	CVD	5.7
		Ashur S T et al., 2015 [59]	Malaysia, UMIC	Single	Validation study	Hospital based	125	T2DM	–
		Moharamzad Y et al., 2015 [109]	Iran, LMIC	Multicentric	Validation study	Hospital based and Community based	200	Hypertension	54
		Tandon S et al., 2015 [135]	Africa, UMIC	Single	Validation study	Hospital based	154	T2DM	35.71
		Anbazhakan S et al., 2016[56]	India, LMIC	Multicentric	Validation study	Community based	180	T2DM, Hypertension	59.40
		Polanska B J et al., 2016 [97]	Poland, HIC	Single	Validation study	Hospital based	110	Hypertension	24.50
		Okello S et al., 2016 [174]	Uganda, LMIC	Single	Validation study	Hospital based	329	Hypertension	85
		Zongo A et al., 2016 [177]	Canada, HIC	Single	Validation study	Community based	901	T2DM	14.50
		Cabral A C et al., 2017 [64]	Portugal, HIC	Multicentric	Validation study	Hospital &community based	472	Hypertension	28
		Janezic A et al., 2017 [84]	Slovenia, HIC	Multicentric	Validation study	Community based	208	Asthma	24
		Laghousi D et al., 2020 [99]	Iran, LMIC	Single	Validation study	Hospital based	320	T2DM	–
		Martinez-Perez P et al., 2021 [106]	Spain, HIC	Single	Validation study	Hospital based	232	T2DM	–
		Iranpour A et al., 2022 [81]	Iran, LMIC	Single	Validation study	Hospital based	150	T2DM	–
2	GMAS	Naqvi A A et al., 2018 [115]	Pakistan, LMIC	Multicentric	Development	Hospital based	161	Chronic diseases (CVD, Pulmonary diseases, CNS, GI diseases)	–
		Naqvi A A et al., 2019 [116]	Saudi Arabia, HIC	Multicentric	Validation study	Hospital based	171	Chronic diseases (T2DM, hypertension,..)	–
		Naqvi A et al., 2019 [117]	Pakistan, LMIC	Multicentric	Validation study	Hospital based	196	Chronic diseases(Hypertension, T2DM, COPD, Asthma,..)	–
		Naqvi A A et al., 2020 [175]	Saudi Arabia, HIC	Multicentric	Validation study	Hospital based	282	Chronic diseases (Hypertension, T2DM,..)	38.30
		Mahmoud M A et al., 2021 [101]	Sudan, LMIC	Single	Validation study	Hospital based	500	T2DM	–
		Nguyen T H et al., 2021 [118]	Vietnam, LMIC	Single	Validation study	Hospital based	165	T2DM	–
		Shrestha R et al., 2021 [130]	Nepal, LMIC	Multicentric	Validation study	Hospital based and community based	220	Chronic diseases (Hypertension, T2DM,..)	–
		Wang Y et al., 2021 [148]	China, UMIC	Multicentric	Validation study	Hospital based and community based	312	Chronic diseases (DM, Hypertension, CAD, Asthma, COPD,..)	–
		Islam M A et al., 2023 [82]	Jordan, LMIC	Single	Validation study	Hospital based	119	T2DM	33.6
		Islam M A et al., 2023 [83]	Pakistan, LMIC	Single	Validation study	Hospital based	150	Chronic diseases (DM, hypertension)	80.70
		Maryem A et al., 2023 [102]	Morocco, LMIC	Multicentric	Validation study	Hospital based	284	T2DM	–
3	BMQ	Horne R et al., 1999 [46]	UK, HIC	Multicentric	Development	Hospital based	524	Chronic disease (Asthma, T2DM,..)	–
		Svarstad B L et al., 1999 [133]	USA, HIC	Single	Validation study	Community based	43	Hypertension	–
		Alhalaiqa F et al., 2014 [54]	Jordan, LMIC	Multicentric	Validation study	Hospital based	605	Chronic diseases	–
		Jimenez K et al., 2016 [86]	USA, HIC	Single	Validation study	Community based	73	T2DM	Unintentional: 61 Intentional: 75 Cost-related: 26
		Mostafavi F et al., 2016 [155]	Iran, LMIC	Multicentric	Validation study	Community based	612	Hypertension	–
		Arikan H et al., 2018[58]	Turkey, LMIC	Single	Validation study	Hospital based	100	Asthma, COPD	–
		Tan C S et al., 2018 [134]	Malaysia, UMIC	Multicentric	Validation study	Community based	238	Hypertension	–
		Ranasinghe P et al., 2018 [124]	Sri Lanka, LMIC	Single	Validation study	Hospital based	165	T2DM	60.60
		Cai Q et al., 2019 [120]	China, UMIC	Single	Validation study	Hospital based	217	Asthma	49.80
		Karbownik MS et al., 2019 [88]	Poland, HIC	Single	Validation study	Hospital based	311	CVD	–
4	ARMS	Kripalani S et al., 2009 [93]	Georgia, UMIC	Single	Development	Hospital based	435	CHD	–
		Mayberry L S et al., 2013 [107]	USA, HIC	Single	Validation study	Hospital based	314	T2DM	–
		Kim C J et al., 2016 [91]	Korea, HIC	Single	Validation study	Hospital based	304	T2DM	–
		Gokdogan F et al., 2017 [77]	Turkey, LMIC	Multicentric	Validation study	Hospital based	100	Chronic diseases (Hypertension, T2DM, CHF,..)	–
		Lomper K et al., 2018 [152]	Poland, HIC	Single	Validation study	Hospital based	279	Chronic diseases (CAD, hypertension, T2DM,..)	48.40
		Chen Y J et al., 2020 [181]	China, UMIC	Single	Development	Community based	120	Hypertension	85.70
		Alammari G et al., 2021 [53]	Saudi Arabia, HIC	Single	Validation study	Hospital based	264	Chronic diseases (Hypertension, T2DM, CVD,..)	63.86
5	MGLS/ MMAS 4	Morisky D E et al., 1986 [112]	USA, HIC	Multicentric	Development	Hospital based	400	Hypertension	–
		Prado J C et al., 2007 [178]	Brazil, UMIC	Single	Validation study	Community based	109	Hypertension	62.10
		Wang Y et al., 2012 [145]	Singapore, HIC	Multicentric	Validation study	Community based	294	T2DM	–
		Kristina S A et al., 2019 [94]	Indonesia, UMIC	Multicentric	Validation study	Community based	250	T2DM	24.80
		Llorca C V et al., 2020 [143]	Spain, HIC	Multicentric	Validation study	Community based	6237	Chronic diseases (Hypertension, T2DM,..)	–
		Mehrabi S et al., 2023 [108]	Iran, LMIC	Single	Validation study	Hospital based	62	Asthma	29.03
6	HBCTS	Kim M T et al., 1999 [89]	USA, HIC	Multicentric	Development	Community based	Study 1: 139, Study 2: 341	Hypertension	–
		Dehghan M et al., 2014 [50]	Iran, LMIC	Single	Validation study	Hospital based	280	Hypertension	–
		Cheong AT et al., 2015 [72]	Malaysia, UMIC	Multicentric	Validation study	Hospital based	299	Hypertension	–
		Uchmanowicz I et al., 2016 [139]	Poland, HIC	Single	Validation study	Hospital based	117	Hypertension	–
		Pan J et al., 2020 [119]	China, UMIC	Single	Validation study	Hospital based	234	Hypertension	–
		Shakya R et al., 2022 [127]	Nepal, LMIC	Multicentric	Validation study	Community based	282	Hypertension	89.40
7	TSQM	Atkinson M J et al., 2004 [161]	USA, HIC	Single	Development	Community based	567	Chronic diseases (asthma, CVD, T2DM,...)	–
		Bharmal M et al., 2009 [63]	USA, HIC	Web based	Validation study	Community based	396	Hypertension	–
		Liberato A C S et al., 2016 [104]	Brazil, UMIC	Single	Validation study	Hospital based	190	CHD	–
		Liberato A C S et al., 2020 [96]	Brazil, UMIC	Single	Validation study	Hospital based	300	Hypertension	10.20
8	TSQM Version II	Atkinson M J et al., 2005 [162]	USA, HIC	Multicentric	Validation study	Community based	342	Chronic diseases (asthma, CVD, T2DM,..)	–
9	MARS-5	Mahler C et al., 2010 [105]	Germany, HIC	Single	Validation study	Hospital based	Study 1: 241 Study 2: 282	Chronic diseases (CVD, hypertension hyperlipidemia)	–
		Ladova K et al., 2014 [98]	Czech Republic, HIC	Single	Validation study	Hospital based	136	CV risk and LDL-c goal	7.40
		Tommelein E et al., 2014 [138]	Belgium, HIC	Multicentric	Validation study	Community based	613	COPD	47.10
		Chan A H Y et al., 2019 [67]	south-west London, HIC	Multicentric	Development	Community based	Hypertension (A, B)= 228 (50, 178), T2DM =100, Asthma=100	Hypertension, DM, Asthma	–
10	SEAMS	Risser J et al., 2007 [123]	Georgia, UMIC	Single	Development	Hospital based	436	CHD	–
		Wu J et al., 2020 [151]	China, UMIC	Single	Validation study	Hospital based	204	T2DM	–
		Alhazzani H et al., 2021 [55]	Saudi Arabia, HIC	Single	Validation study	Hospital based	264	Chronic diseases (DM, hypertension asthma, COPD, and CVD)	–
11	MALMAS	Chung W W et al., 2014 [74]	Malaysia, UMIC	Single	Validation study	Hospital based	136	T2DM	–
		Chung W W al., 2014 [73]	Malaysia, UMIC	Single	Validation study	Hospital based	136	T2DM	–
		Lai P SM et al., 2020 [100]	Malaysia, UMIC	Single	Validation study	Hospital based	100	T2DM	–
12	MARS-10	Cohen J L et al., 2009 [49]	USA, HIC	Multicentric	Validation study	Hospital based	318	Asthma	69.40
		Mora P A et al., 2011 [111]	USA, HIC	Multicentric	Validation study	Hospital based	294	Asthma	–
		Tangirala N C et al., 2020 [136]	USA, HIC	Multicentric	Validation study	Hospital based	Asthma: 452COPD: 393	Asthma, COPD	–
13	MAR- Scale	Unni E J et al., 2009 [142]	USA, HIC	Single	Development	Community based	Cholesterol-lowering medications: 420, asthma maintenance medications: 399	CVD, Asthma	–
		Unni E J et al., 2014 [141]	USA, HIC	Single	Validation study	Hospital based	350	Asthma, Hypercholesterolemia	Cholesterol lowering medications: 25.5, Asthma maintenance medications: 48.75
		Shima R et al., 2015 [129]	Malaysia, UMIC	Multicentric	Validation study	Community based	665	Hypertension	–
14	HBCS	Karademir M et al., 2009 [87]	Turkey, LMIC	Multicentric	Validation study	Community based	200	Hypertension	Unintentional: 33.3 Intentional: 14.6
		Nakwafila O et al., 2022 [173]	Africa, UMIC	Multicentric	Validation study	Community based	400	Hypertension	12.30
15	KWood-MAS-4/ Low Pharmacy Refill Adherence	Krousel-Wood M et al., 2013 [169]	USA, HIC	Single	Development	Community based	394	Hypertension	23.40
		Krousel-Wood M et al., 2019 [170]	USA, HIC	Single	Validation study	Community based	1532	CHD, hypertension	38.7
16	MASES	Ogedegbe G et al., 2003 [156]	USA, HIC	Single	Development	Community based	Item generation = 106, Item analysis = 72	Hypertension	–
		Saffari M et al., 2015 [125]	Iran, LMIC	Multicentric	Validation study	Community based	184	Hypertension	–
17	MASES-SF	Hacihasanoglu R et al., 2012 [70]	Turkey, LMIC	Single	Validation study	Community based	150	Hypertension	–
18	MASES- R	Fernandez S et al., 2008 [180]	Columbia, UMIC	Single	Validation study	Hospital based	168	Hypertension	–
19	MAQ for asthma	Axelsson M et al. 2016 [164]	West Sweden, HIC	Single	MA assessment & validation	Community based	700 Phase 1:300 Phase 2:200 Phase 3:200	Asthma	–
20	MAQ for DM	Anuradha HV et al., 2022 [57]	India, LMIC	Single	Development	Hospital based	30	T2DM	–
21	M-DRAW	Lee S et al., 2017 [95]	USA, HIC	Single	Validation study	Community based	26	Chronic diseases (Hypertension, Dyslipidemia, T2DM and Chronic pain)	–
		Lee S et al., 2019 [171]	USA, HIC	Single	Validation study	Community based	88	T2DM	–
22	TAI	Plaza V et al., 2016 [122]	Spain, HIC	Multicentric	Development	Hospital based	1009	Asthma or COPD	62.50
		Muneswarao J et al., 2020 [113]	Malaysia, UMIC	Single	Validation study	Hospital based	120	Asthma	–
23	TAQPH	Ma C et al., 2011 [153]	China, UMIC	Multicentric	Development	Hospital based	278	Hypertension	–
		Dehghan M et al., 2015 [62]	Iran, LMIC	Multicentric	Validation study	Hospital based	330	Hypertension	–
24	ASK-20	Hahn S R et al., 2008 [48]	USA, HIC	Web based	Development	Community based	605 (Asthma - 200, Depression - 202, and T2DM - 203)	Chronic diseases (asthma, T2DM,..)	–
		Matza L S et al., 2008 [159]	USA, HIC	Single	Validation study	Hospital based	112	Asthma, T2DM, or CHF	–
25	ASK-12	Matza L S et al., 2009 [160]	USA, HIC	Single	Validation study	Hospital based	112	Chronic diseases (Asthma, T2DM, CHF)	–
26	LMAS	Bou Serhal R et al., 2018 [167]	Lebanon, LMIC	Multicentric	MA assessment & validation	Hospital based	405	Hypertension	17.60
		Ibrahim L et al., 2020 [80]	Lebanon, LMIC	Multicentric	Validation study	Community based	182	T2DM	42.80
27	DMAS	Ayoub D et al., 2019 [165]	Lebanon, LMIC	Multicentric	Development	Hospital based	500	T2DM	66.20
		Mallah Z et al., 2019 [172]	Lebanon, LMIC	Multicentric	Validation study	Hospital based	300	T2DM	66.30
28	IADMAS	Mikhael E M et al., 2019 [110]	Iraq, UMIC	Single	Development	Hospital based	80	T2DM	–
29	SPUR - 27	Wells J et al., 2023 [150]	UK, HIC	Single	Validation study	Hospital based	100	COPD	–
30	SPUR 45	Wells J S et al., 2022 [149]	UK, HIC	Single	Validation study	Hospital based	378	T2DM	–
31	A 14 scale	Jank S et al., 2009 [85]	Germany, HIC	Single	Development	Hospital based	149	Chronic diseases (Hypertension, T2DM,..)	40
32	A 12-item Medication Adherence Scale	Ueno H et al., 2018 [140]	Japan, HIC	Single	Development	Hospital based	540	Chronic diseases (Hypertension, T2DM, CVD,..)	–
33	AAMQ-13	Nassar R I et al. 2022 [114]	Jordan, LMIC	Single	Development	Hospital based	213	Asthma	–
34	MeDS	Bailey S et al., 2015 [61]	USA, HIC	Single	Development	Hospital based	193	T2DM, Hypertension	–
35	MAUQ	Cabral A C et al., 2023 [65]	Portugal, HIC	Multicentric	Development	Community based	300	Hypertension	–
36	MPRAQ	Chan A H Y et al., 2021 [66]	UK, Netherlands, HIC	Multicentric	MA assessment & validation	Community based	Face validity: 15, mTurk:184, COPA: 334	Chronic diseases (T2DM, asthma, COPD, CVD, malignant)	–
37	MyMAAT-12	Hatah E et al., 2020[79]	Malaysia, UMIC	Multicentric	Development	Hospital based and Community based	495	T2DM	63.80
38	FATS	Fongwa M N et al., 2015 [75]	USA, HIC	Single	Validation study	Hospital based	147	Hypertension	–
39	GAS	Shi Z et al., 2021 [128]	China, UMIC	Multicentric	Validation study	Hospital based	336	T2DM	–
40	IAQ	Toelle B G et al., 2020 [137]	Australia, HIC	Multicentric	Validation study	Hospital based	74	Asthma	59.50
41	ChMAR-Scale	Chen P F et al., 2020 [71]	Taiwan, HIC	Multicentric	Validation study	Hospital based and Community based	538	Hypertension	61.60
42	MMWFU	Weinman J et al., 2019 [146]	UK, HIC	Multicentric	Development	Community based	145	T2DM	–
43	IMAS	Wang Y H et al., 2023 [147]	Taiwan, HIC	Single	Development	Hospital based	235	COPD	–
44	PSAM questionnaire	Mathias S D et al., 2001 [154]	USA, HIC	Single	Development	Hospital based	53	Asthma	–
45	MUAH questionnaire	Wetzels G et al., 2006 [182]	Netherlands, HIC	Multicentric	Development	Community based	255	Hypertension	–
46	HBMA scale	Song Y et al., 2011 [131]	USA, HIC	Single	Validation study	Community based	525	Hypertension	Study 1: 60.6 Study 2: 54.6
47	SATMED-Q	Ruiz M A et al., 2008 [158]	Spain, HIC	Multicentric	Development	Hospital based	455	Chronic diseases (T2DM, Hypertension, asthma, COPD,..)	–
48	MCQ	Fadhilah AN et al., 2019 [163]	Malaysia, UMIC	Single	MA assessment & validation	Hospital based	232	T2DM	44.80
49	PDSMS	Wallston K A et al., 2007 [179]	USA, HIC	Multicentric	Development	Hospital based	398 (Type 1: 57 Type 2: 341)	Type 1 and T2DM	–
50	BBQ	George J et al., 2005 [47]	Australia, HIC	Single	Development	Community based	276	COPD	–
51	ASCD	Buszko K et al., 2016 [45]	Poland, HIC	Single	Validation study	Hospital based	413	CVD	–
52	ITBQ	Munoz Cobos F et al., 2024 [43]	Spain, HIC	Multicentric	Validation study	Community based	262	COPD	–
53	SMAQ	Soares S M et al., 2024 [44]	Spain, HIC	Multicentric	Validation study	Hospital based	117	Hypertension	79.50
55	HBCS, A 14 scale	Chatziefstratiou A et al., 2019 [68]	Greece, HIC	Single	Validation study	Hospital based	68	Hypertension	–
56	MMAS 4, BMQ	Ben A J et al., 2012 [166]	Southern Brazil, UMIC	Multicentric	Validation study	Community based	206	Hypertension	–
57	MMAS 4, HBCS	Koschack J et al., 2010 [92]	Germany, HIC	Single	Validation study	Hospital based	353	Hypertension	MMAS 4:39 HBCS: 36
58	MMAS 4, MARS-5	Van de Steeg N et al., 2009 [144]	Germany, HIC	Single	Validation study	Community based	128	Hypertension	MMAS 4: 28.9MARS-5: 4.8
59	MARS-5, BMQ	Al Qerem W et al., 200022 [52]	Jordan, LMIC	Single	Validation study	Hospital based	485	Chronic diseases (Hypertension, T2DM, CVD, Asthma)	–
60	MMAS-8, VAS	Gallagher B D et al, 2014 [76]	USA, HIC	Multicentric	Validation study	Hospital based	149	Hypertension	–
61	MMAS 8, SR - 4	Zongo A et al., 2015 [176]	Canada, HIC	Single	Validation study	Community based	156	T2DM	SR- 4:0.6 MMAS 8: 13.10
63	HBM -based questionnaire, BMQ	Tordera M P et al., 2009 [157]	Spain, HIC	Single	Validation study	Community based	126	Asthma	–
64	PDSMS, MUSE, MMAS-8	Al abboud S A et al., 2016 [69]	Malaysia, UMIC	Single	Validation study	Hospital based	62	T2DM	–

^a^Clinical trials: Experiments or observations designed to answer specific questions about interventions; Hospital based: Patients evaluated by their doctors or physicians at the hospitals or clinics during the routine care; Community based: Patients evaluated by their doctors or physicians at the community setting.

**Abbreviations: MA** – Medication adherence, **HIC** – High Income Country, **UMIC**-Upper Middle Income Country, **LMIC**- Lower Middle Income Country, **USA**- United States of America, **UK**- United Kingdom, **BMQ** - Beliefs About Medication Questionnaire, **MARS-5** -Medication Adherence Report Scale, **ARMS** - Adherence to Refills and Medications Scale, **SEAMS** - Self-Efficacy for Appropriate Medication Use Scale, **MMAS-8** - eight-item Morisky Medication Adherence Scale, **COPD** - Chronic obstructive pulmonary disease, **MeDS**- Measure of Drug Self-Management, **DMAS**- Diabetes Medication Adherence Scale, **TAQPH**- Treatment Adherence Questionnaire for Patient with Hypertension, **TSQM**- Treatment Satisfaction Questionnaire for Medication, **MAUQ**- Medication Adherence Universal Questionnaire, **MPRAQ**- Medication Practical barriers to Adherence Questionnaire, **PDSMS**- Perceived Diabetes Self-Management Scale, **MUSE**- Medication Understanding and Use Self Efficacy Scale, **GMAS**- General Medication Adherence Scale, **MASES-SF**- Medication Adherence Self-Efficacy Scale-Short Form, **HBCTS**- Hill Bone compliance to High Blood Pressure Therapy Scale, **MALMAS**- Malaysian Medication Adherence Scale, **VAS**- Visual Analogue Scale, **M-DRAW** - Modified Drug Adherence Work-up Tool, **DMAS** - Diabetes Medication Adherence Scale, **TAI**- Test of Adherence to Inhalers, **HBCS** – Hill Bone compliance scale, **MASES**- Medication Adherence Self-efficacy Scale, **GAS-C** - General Adherence Scale in Chinese, **MAR-Scale** - Medication Adherence Reasons Scale, **HBMA**- Hill-Bone Medication Adherence scale, **IAQ**- Inhaler Adherence Questionnaire, **MGL**- Morisky–Green–Levine, **ASK-20** - Adherence Starts with Knowledge, **MGT**- Morisky-Green test, **ICS –** Inhaled corticosteroids, **K Wood-MAS-4-**4-item Krousel-Wood Medication Adherence Scale, **PSAM**- Patient Satisfaction with Asthma Medication, **T2DM –** Type 2 Diabetes Mellitus, **NR**- Not Reported, **LDL-** Low-density lipoprotein, **MAQ-** Medication Adherence Questionnaire, **LMAS-**Lebanese Medication Adherence Scale, **IADMAS-** Iraqi Anti-Diabetic Medication Adherence Scale, **AAMQ-** Adherence to Asthma Medication Questionnaire, **MyMAAT-** Malaysia Medication Adherence Assessment Tool, **ChMAR-Scale-** Chinese version of Medication Adherence Reasons Scale, **MMWFU-** Making Medicines Work For You, **IMAS-** Inhaled Medication Adherence Scale, **MUAH-**Maastricht Utrecht Adherence in Hypertension**, SATMED-Q-**Treatment Satisfaction with Medicines Questionnaire, **MCQ-**Medication Compliance Questionnaire, **BBQ-** Beliefs and Behavior Questionnaire, **CVD**- Cardiovascular Disease, **CAD**- Coronary Artery Disease, **CHD**- Congestive Heart Disease, **CHF**- Congestive Heart Failure, **FATS-** Facilitators of and Barriers to Adherence to Hypertension Treatment Scale**, SR-** self-report**, HBM-** Health belief model, **ASCD**- Adherence Scale in Chronic Diseases, **ITBQ**- Inhaled Therapy Beliefs Questionnaire, **SMAQ**- Simplified Medication Adherence Questionnaire

**Table 2:** Psychometric properties of scales with the methods and standards from developmental studies.

Self-report adherence Scale	Author, Year	Response rate (%)	Internal consistency	Sensitivity (%)	Specificity (%)	Stable over time (Pearson’s/ Spearman’s)	Correlation (Criterion validity)	Validity	Methods/Standards against which validity assessed	Any other measure	Stage of medication taking
MMAS 4/ MGT/ MAQ	Morisky D E et al., 1986 [112]	–	α = 0.61	81	44	–	Significant	Reliability	Internal consistency	–	Implementation, Discontinuation
								Criterion- related Validity (concurrent)	BP		
								Construct validity	PCA		
BMQ	Horne R et al., 1999 [46]	83%	α= 0.600.83	–	–	–	Significant	Reliability	Internal consistency Test-retest	–	?
								Criterion-related validity	BMQ subscales		
								Construct (discriminant) validity	PCA, CFA		
HBCTS	Kim M T et al., 1999 [89]	–	Study 1: α = 0.74, Study 2: α = 0.84	–	–	–	?	Reliability	Internal consistency	–	Implementation
								Content validity	CVI		
								Construct validity	PCA, Factor analysis		
								Predictive validity	BP		
PSAM	Mathias S D et al., 2001 [154]	–	α = 0.820.88	–	–	–	Significant	Reliability	Internal consistency, Test-retest	ICC = 0.70	?
								Content validity			
								Criterion- related validity	PSAM Subscales		
								Construct validity	Pearson’s correlation coefficient, Spearman’s rank correlation		
MASES	Ogedegbe G et al., 2003 [156]	–	α = 0.96	–	–	–	Significant	Reliability	Internal consistency, Test-retest	–	Implementation
								Criterion-related validity	BP		
TSQM	Atkinson M J et al., 2004 [161]	67.2	α = 0.85–0.87	–	–	–	?	Reliability	Internal consistency	–	?
								Construct validity	EFA		
TSQM Version II	Atkinson M J et al., 2005 [162]	81.4	–	–	–	–	Significant	Reliability	Internal consistency	CFI = 0.98	?
								Criterion (Concurrent) validity	Regression and discriminant analytic models		
								Construct validity	Structural equation modeling, PCEFA		
BBQ	George J et al., 2005 [47]	75	α = 0.62–0.94	–	–	–	Significant	Reliability	Internal consistency	–	?
								Criterion validity	MARS		
								Construct (convergent, discriminant) validity	PCA, Correlation with MARS		
								Face validity			
								Content validity			
MUAH	Wetzels G et al., 2006 [182]	80 - 98	I, α = 0.75, II, α = 0.80, III, α = 0.63 and IV, α = 0.76	–	–	–	?	Reliability	Internal consistency Test-retest	I) ICC = 0.86, II) ICC = 0.80, III) ICC = 0.85 and IV) ICC =0.79	?
								Construct (Convergent) validity	Association between MEMS, BMQ and pharmacy refill records, PCA		
SEAMS	Risser J et al., 2007 [123]	–	α = 0.89	–	–	Spearman’s ρ = 0.57, p = 0.0001	Significant	Reliability	Internal consistency Test-retest	–	?
								Criterion-Related Validity	MMAS 4/ MGT, BP		
PDSMS	Wallston K A et al., 2007 [179]	–	α = 0.834	–	–	–	Significant	Reliability	Internal consistency	ITC = 0.3900.707	?
								Construct validity	PCA, EFA		
ASK-20	Hahn S R et al., 2008 [48]	88.3	α = 0.85	–	–	–	?	Reliability	Internal consistency	–	Implementation, Discontinuation
								Construct (convergent) validity	HbA1c, blood glucose meter readings		
								Content validity			
SATMED-Q	Ruiz M A et al., 2008 [158]	96.7	α = 0.82	–	–	–	Significant	Reliability	Internal consistency	ICC = 0.943, GFI = 0.938, AGFI = 0.909, CFI = 0.860, RMR = 0.069 and RMSEA = 0.053	?
								Content validity			
								Construct validity	CFA		
								Discriminant validity			
								Criterion-related (concurrent) validity	MMAS 4, TSQM		
A 14 scale	Jank S et al., 2009 [85]	55.3	α =0.861	–	–	Spearman’s rho, ρ = 0.43	Significant	Reliability	Internal consistency	Nil	?
								Content validity			
								Criterion-related validity	MMAS 8		
ARMS	Kripalani S et al., 2009 [93]	–	α = 0.814	–	–	Spearman’s rho = -0.651, P < 0.01	Significant	Reliability	Internal consistency Test-retest	ICC = 0.250	Implementation, Discontinuation
								Criterion-related validity	MMAS 4, Subscales, BP, Medication refill adherence		
								Predictive validity	Medication refill adherence		
MAR-Scale	Unni E J et al., 2009 (Cholesterol-lowering medications)[142]	–	α = 0.616 to 0.752	–	–	Correlation coefficient = 0.495	?	Reliability	Internal consistency Test-retest	–	Implementation, Discontinuation
								Face validity			
								Construct (Convergent) validity	MMAS 4/ MGT		
MAR-Scale	Unni E J et al., 2009 (asthma maintenance medications)[142]	–	α = 0.654 to 0.881	–	–	Correlation coefficient = 0.481	?	Reliability	Internal consistency Test-retest	–	Implementation, Discontinuation
								Face validity			
								Construct (Convergent) validity	MMAS 4/ MGT		
TAQPH	Ma C et al., 2011 [153]	–	α = 0.86	–	–	–	Significant (all)	Reliability	Internal consistency Test-retest	Bartlett’s test, v2 = 7297.33, P < 0.001, KMO = 0.83, ICC = 0.82	?
								Content validity			
								Construct validity	EFA, CFA		
								Criterion-related validity	MMAS 4 and GSES score		
K Wood-MAS-4/ Low Pharmacy Refill Adherence	Krousel -Wood M et al., 2013 [169]	–	–	67.4	67.8	–	–	Predictive validity	MMAS-8and HBCS	–	?
MeDS	Bailey S et al., 2015 [61]	88.6	α = 0.72	–	–	–	Significant (all)	Reliability	Internal consistency	–	?
								Construct validity	MMAS 8		
								Criterion- related validity	LDL, DBP		
MALMAS	Chung W W et al., 2015 [73]	–	α = 0.565	88.9	29.6	Spearman’s rho = 0.412 (p<0.001)	Significant (all)	Reliability	Internal consistency Test-retest	–	?
								Criterion-related(Concurrent) validity	HbA1c		
								Construct (Convergent) validity	MMAS 8		
Medication Adherence Questionnaire for Asthma	Axelsson M et al. 2016 [164]	52	α = 0.8800.927	–	–	–	–	Reliability	Internal consistency	–	?
								Construct validity	MARS		
TAI	Plaza V et al., 2016 [122]	–	α = 0.86	67.4	66	–	Significant	Reliability	Internal consistency Test-retest	KMO = 0.905, ICC = 0.883	?
								Construct validity	PFA		
								Criterion validity	MMAS 4/ MGT, Electronic adherence, ACT, CAT		
LMAS	Bou Serhal R et al., 2018 [167]	–	–	82.9	36.9	–	Significant	Criterion-related (Concurrent) validity	MMAS 8	KMO coefficient = 0.743, ICC average measure = 0.651	?
A 12-item Medication Adherence Scale	Ueno H et al., 2018 [140]	60.7	α = 0.78	–	–	–	–	Reliability	Internal consistency	CFI = 0.94 and RMSEA = 0.069	?
								Construct validity			
DMAS	Ayoub D et al., 2019 [165]	–	α = 0.612	70.39	51.47	Spearman’s rho = 0.699, P < 0.001	Significant	Reliability	Internal consistency Test-retest	Cohen’s kappa = 0.566, KMO measure was 0.705 and the Bartlett test was significant (P < 0.001)	?
								Criterion- related validity	LMAS-14		
								Construct (Convergent) validity,	LMAS-14 score and DMAS		
MARS-5	Chan A H Y et al., 2019 [67]	71-100	α = 0.67–0.89	–	–	Hypertension A (r =0.97, P <.001)	Significant	Reliability	Internal consistency Test-retest	–	Implementation, Discontinuation
								Face validity			
								Criterion-related Validity	BP		
								Construct validity	BMQ		
IADMAS	Mikhael E M et al., 2019 [110]	95.2	α = 0.712	100	33.9	Spearman’s rho = 0.806 (p=0.016)	Significant	Reliability	Test-retest	–	?
								Face validity			
								Content validity			
								Construct (Convergent) validity	MAQ		
								Criterion-related (Concurrent) validity	HbA1c		
GMAS	Naqvi A A et al., 2018 [115]	91	α = 0.84	> 74	–	Pearson’s coefficient = 0.996 (p-value < 0.01)	–	Reliability	Test-retest	CVI =0.8 (SD 0.147); McDonald’s coefficient, F1 = 0.86, F2= 0.9, F3= 0.75; ICC, F1 =0.806 (0.775–0.835), F2 = 0.778 (0.741–0.811), F3 = 0.445 (0.326–0.542), NFI = 0.95, TLI = 0.92, and CFI = 0.96, RMSEA = 0.06 and SRMR = 0.03	?
								Face validity			
								Content validity			
								Construct (Convergent and discriminant) validity			
								Known group validity	Subscales		
MMWFU	Weinman J et al., 2019 [146]	90.1	–	Category 1: 69.5 Category 2:100.0	Category 1: 68.3 Category 2: 27.0	–	–	Construct (Convergent) validity	MMAS 4, BMQ	–	?
ChMAR-Scale	Chen P F et al., 2020 [71]	86.6	α = 0.649 to 0.852	–	–	–	Significant	Reliability	Internal consistency	–	?
								Criterion-related validity	VAS		
								Construct (Convergent) validity			
ARMS-C	Chen Y J et al., 2020 [181]	–	α = 0.89	–	–	–	–	Reliability	Internal consistency Test-retest	KMO = 0.79, Bartlett’s test = χ =707.3, p<0.001, eigenvalue of F1 = 1.91, eigenvalue of F2 = 1.31, ICC = 0.86 (p<0)	Implementation, Discontinuation
								Construct validity	Factor analysis		
MyMAAT-12	Hatah E et al., 2020 [79]	–	α = 0.91	Using HbA1c: 72.9, MPR:82.7 and pharmacist’s subjective assessment:77.7	Using HbA1c: 43, MPR: 39.5 and pharmacist’s subjective assessment:49.2	Spearman’s ρ = 0.44, p-value <0.001	–	Reliability	Internal consistency Test-retest	I-CVI=1, S-CVI = 1, KMO = 0.92, χ2= 5604.1, < 0.001, ICC = 0.97 (95% CI 0.93 to 0.98)	?
								Face validity			
								Content validity			
								Linguistic validity			
								Construct (Convergent) validity	SEAMS		
MPRAQ	Chan A H Y et al., 2021 [66]	COPA: 50.2%	α = 0.89 (mTurk) α = 0.94 (COPA)	–	–	BMQ specific: Concerns (mTurk r = 0.546, P <.0001; COPA r = 0.370, P = <.0001); necessity beliefs (mTurk, r = 0.205, P =.005); BMQ general: BMQ-Overuse (mTurk r = 0.324, P <.0001; COPA r = 0.109, P =.047) and Harms subscales (mTurK r = 0.504, P <.0001; COPA r = 0.219, P <.0001); MARS-5: (mTurk r = −0.450, P <.0001; COPA r = −0.260, P <.0001); PSM|: mTurk sample (r = 0.463, P <.0001)	–	Reliability	Internal consistency Test-retest	–	?
								Face validity			
								Construct validity	BMQ, MARS-5, PSM		
								Discriminant validity	Acceptability questionnaire		
Medication Adherence Questionnaire for DM	Anuradha HV et al., 2022 [57]	–	α = 0.927	–	–	Spearman’s rho = 0.91	–	Reliability	Internal consistency	–	?
								Content validity			
AAMQ-13	Nassar R I et al. 2022 [114]	–	α = 0.87	84.8	95.2	Spearman-Brown coefficient= 0.743	Significant	Reliability	Internal consistency	Variance = 51.76%	?
								Face validity			
								Content validity			
								Criterion-concurrent validity	TAI, pharmacy refill records		
								Construct validity (convergent)	ACT questionnaire and PHBS		
MAUQ	Cabral A C et. al., 2023 [65]	–	α = 0.569	–	–	–	–	Construct (Convergent) validity	MUAH-16	–	?
IMAS	Wang Y H et al., 2023 [147]	–	α = 0.81 to 0.95	–	–	–	–	Reliability	Internal consistency, composite reliability	CVI = 0.981 (Relevance), CVI = 0.987 (Clarity), CFI=1.00, TLI=1.00, RMSEA=0.00, and SRMR=0.06	?
								Content validity			
								Construct validity			

**Abbreviations: MMAS 4 -**Morisky Medication Adherence Scale – 4, **MGT**-Morisky-Green test, **MGLS-** Morisky Green Levine Adherence Scale, **MAQ-** Medication Adherence Questionnaire, T2**DM-** Diabetes Mellitus, **MeDS-** Measure of Drug Self-Management, **LDL-** Low-density lipoprotein, **IADMAS-** Iraqi Anti-Diabetic Medication Adherence Scale, **DMAS-** Diabetes Medication Adherence Scale, **LMAS-14-** Lebanese Medication Adherence Scale, **MARS-**Medication Adherence Report Scale, **DBP-**diastolic blood pressure, **KMO-**Kaiser–Meyer–Olkin test, **MAUQ -**Medication Adherence Universal Questionnaire, **MUAH-16-**Maastricht Utrecht Adherence in Hypertension, **MPRAQ-** Medication Practical barriers to Adherence Questionnaire, **BMQ-** Beliefs about Medicines Questionnaire, **PSM-** Perceived Sensitivity to Medicines questionnaire, **MyMAAT-12-** Malaysia Medication Adherence Assessment Tool, **MPR-**Medication Possession ratio, **SEAMS-**Self-Efficacy for Appropriate Medication Use Scale, **CVI-**Content validity index, **ICC -** Intra-class correlation coefficient, **NFI -** Normed fit index, **TLI -** Tucker– Lewis index, and **CFI -** Comparative fit index, **RMSEA -** Root mean square error of approximation, **ACT-**Asthma Control Test questionnaire, **PHBS-** Positive Health Behaviors Scale, **MCQ-**Medication Compliance Questionnaire, **MAQ-**Medication Adherence Questionnaire, **OHA-**Oral Hypoglycemic agents, **GSES-**General Self Efficacy Scale, **PDSMS-** Perceived Diabetes Self-Management Scale, **MALMAS-**Malaysian Medication Adherence Scale, **ARMS-** Adherence to Refills and Medications Scale, **GMAS-** General Medication Adherence Scale, **MMWFU-** Making Medicines Work For You, **TAQPH-** Treatment Adherence Questionnaire for Patient with Hypertension, **TSQM-** Treatment Satisfaction Questionnaire for Medication, **ChMAR-Scale-** Chinese version of Medication Adherence Reasons Scale, **HBCTS-** Hill-Bone compliance to high blood pressure therapy scale, **MASES-** Medication Adherence Self-Efficacy Scale, **VAS-** Visual Analog Scale, **K Wood-MAS-4-**4-item Krousel-Wood Medication Adherence Scale, **MAR-Scale-** Medication Adherence Reasons Scale, **BBQ-** Beliefs and Behavior Questionnaire, **TAI-** Test of Adherence to Inhalers, **AAMQ-13-** Adherence to Asthma Medication Questionnaire -13, **PSAM-** Patient Satisfaction with Asthma Medication, **IMAS-** Inhaled Medication Adherence Scale, mTurk- Amazon mechanical Turk, **COPA-** Consumer panel from the Netherlands, **SATMED-Q-**Treatment Satisfaction with Medicines Questionnaire, **ASK-20-** Adherence Starts with Knowledge, **BP-** Blood Pressure, **HBCS-** Hill Bone Compliance Scale, **CAT-** COPD Assessment Test, **MEMS-** Medication Event Monitoring system.

**Key findings:** -

1) The scales were developed through a combination of quantitative and qualitative methodologies (focus group discussions, semi-structured interviews, extensive literature review, mixed methods).

2) Response rates documented in 17 studies ranging from 50.2% to 100%.

3) Sensitivity of scales was assessed in 11 studies (28.9%), while specificity in 10 studies (26.3%).

4) Five scales demonstrated high sensitivity, and one scale exhibited high specificity (80–100%).

5) Feasibility was not reported for any of the scales.

6) Cronbach’s alpha reported by 34 studies (89.5%), an acceptable range exceeding 0.7 in 30 studies.

7) High reliability (>0.90) – 3 scales, moderate reliability (0.8–0.9) – 1 scale and insufficient reliability (<0.8) – 9 scales.

8) Ten scales (26.3%) were assessed for their correlation with an objective measure of adherence (one scale evaluated using MEMS, others compared against clinical outcomes).

9) Criterion-related validity – in 56.8% of the scales, revealing significant correlations with various measures including clinical outcomes, electronic adherence measurements, subscales of the current scale, and other self-report scales.

10) Construct validity was undertaken for 73% (n=27) of the scales.

**Table 3:** Medication adherence assessment scale comparison.

Self-report adherence Scale	Developed based on	No. of studies	No. of questions	Time to complete (Minutes)	Cut-off score for adherence	How scale was administered*	Validated in low literacy (Yes/No)	Assess self-efficacy (Yes/No)	Assess reliability (Yes/No)	Stage of medication – taking identified
MMAS 8	MMAS 4	24	8	5−40	≥ 6	Self and researcher administered	Yes	–	Yes	Implementation, Discontinuation
BMQ	Mixed method Literature review, Qualitative interviews with Patients, Health Belief Model and Patient Beliefs	13	10−18	15	−	Self and researcher administered	Yes	Yes	Yes	?
GMAS	Mixed method Literature review and Expert opinion	11	11	10	≥ 27	Self and researcher administered	Yes	−	Yes	?
MMAS 4/ MGT/ MAQ	Literature review: 5−item questionnaire by Green et al.	9	4	−	< 4	Self and researcher administered	No	−	Yes	Implementation, Discontinuation
ARMS	Mixed method Literature review, MAQ and HBCS and Expert panel	7	7−12	10−20	12−20	Self and researcher administered	Yes	−	Yes	Implementation, Discontinuation
MARS-5	Literature review: MARS – 10	6	5	10 − 15	≥20	Self and researcher administered	Yes	−	Yes	Implementation, Discontinuation
HBCTS	Literature review	6	14	5	−	Self and Researcher administered	Yes	Yes	Yes	Implementation
TSQM	Mixed method Literature review, Focus groups, In−depth patient interviews	5	9 − 14	3–6	−	Self−administered	No	−	Yes	?
HBCS	Literature review, HBCS– 14	4	9 − 14	15	−	Self−administered	Yes	Yes	Yes	Implementation
MARS-10	MAQ	3	10	−	≥ 4.5	Self and researcher administered	No	−	Yes	Implementation, Discontinuation
SEAMS	Mixed method SEAMS −21, Literature review, expertise and patient interviews	3	10 − 13	−	−	Self−administered	Yes	Yes	Yes	?
MALMAS	MMAS 8	3	9	5 − 10	6–8	Self and Researcher−administered	Yes	−	Yes	?
LMAS	MMAS 8	2	14	−	38	Researcher−administered	Yes	−	Yes	?
M-DRAW	Not specified	2	13	−	−	Self and researcher administered	No	−	Yes	?
SPUR - 27	original 45−item SPUR	2	27	−	87	Researcher−administered	Yes	−	Yes	?
TAQPH	Mixed method Literature review, focus groups	2	28	−	−	Self−administered	No	−	Yes	?
PDSMS	Not specified	2	8	−	−	Self−administered	Yes	−	Yes	?
A 14-item scale	Literature review: MMAS−4, MARS−5	2	14	−	50 − 56	Self−administered	No	−	Yes	?
DMAS	Literature review	2	7	−	7	Self and Researcher−administered	Yes	No	Yes	?
TAI questionnaire	Mixed method Literature review, Delphi process	2	10 − 12	5.3	50	Self and Researcher−administered	Yes	−	Yes	?
MASES	Mixed method MASES −43 item scale, Patient interviews	2	26	5	−	Self −administered	No	Yes	Yes	Implementation
ASK-20	Mixed method Literature review, patient focus groups and expert panel input	2	20	−	−	Self−administered	No	−	Yes	Implementation, Discontinuation
TSQM Version II	TSQM	1	11	−	−	Self−administered	No	−	Yes	?
MAR-Scale	Mixed method MAR−Scale – 15, Expert opinion, Literature review	1	11 − 20	15	>15	Self −administered	Yes	−	Yes	Implementation, Discontinuation
MUSE	Not specified	1	−	−	−	Self−administered	Yes	−	Yes	?
MASES-SF	MASES	1	13	10 minutes	−	Self−administered	Yes	Yes	Yes	Implementation
MASES- R	MASES	1	13	−	−	Self−administered	No	Yes	Yes	Implementation
FATS	Not specified	1	18	−	−	Self−administered	Yes	−	Yes	?
MCQ	MMAS 4, HBCTS	1	7	−	≥ 27	Self−administered	Yes	−	Yes	?
A 12-item Scale	A 14−item scale	1	12	−	−	Self −administered	Yes	−	Yes	?
VAS	Not specified	1	−	−	−	Self−administered	No	−	Yes	?
GAS	Not specified	1	5	−	−	Researcher−administered	No	−	Yes	?
HBMA scale	HBCS	1	9	5	−	Self −administered	No	−	Yes	?
IAQ	Not specified	1	6	1	−	Self −administered	No	−	Yes	?
SR-4scale	Not specified	1	4	−	> 2	Self −administered	No	−	Yes	?
HBM -based questionnaire	Not specified	1	19	−	−	Self −administered	No	−	Yes	?
ASK -12	ASK−20	1	20	−	−	Self−administered	No	−	Yes	Implementation, Discontinuation
PSAM	Mixed method Literature review: asthma and satisfaction questionnaires, focus groups	1	4	−	−	Self−administered	Yes	−	Yes	?
BBQ	Qualitative method: In-depth interviews, Qualitative interviews with Patients	1	30	8	25	Self-administered	No	–	Yes	?
MUAH	Qualitative method: Semi-structured interviews	1	25	25	–	Self-administered	No	–	Yes	Implementation
SATMED-Q	Mixed method Literature review, Expert opinion, Focus groups	1	17	4.71	–	Self-administered	No	–	Yes	?
MAR-Scale	Mixed method MAR-Scale – 15, Expert opinion, Literature review	1	15	–	> 15	Self-administered	No	–	Yes	?
K Wood-MAS-4/ Low Pharmacy Refill Adherence	Not specified	1	4	< 5	–	Researcher-administered	No	Yes	Yes	?
MeDS	Mixed method In-depth review, Literature review, Expert opinion	1	12	–	–	Self-administered	Yes	No	Yes	?
MAQ for Asthma	Mixed method Literature review, Expertise	1	10	–	–	Self-administered	–	No	Yes	?
MAQ for DM	Not specified	1	12	–	–	Researcher-administered	Yes	No	Yes	?
IADMAS	Literature review: MAQ, MAT and MCQ	1	8	5–10	8	Researcher-administered and Self-administered	Yes	–	Yes	?
MMWFU	Qualitative method: Expert opinion	1	8	–	0-1	Researcher-administered	No	–	Yes	?
ChMAR-Scale	MAR- Scale	1	24	–	–	Researcher-administered	Yes	–	Yes	?
ARMS-C	ARMS 12	1	10	–	<20	Self-administered	No	–	Yes	?
MyMAAT-12	Mixed method Literature review and Expert clinicians	1	12	–	> 54	Researcher- administered	Yes	–	Yes	Implementation
MPRAQ	Literature review	1	15	2-10	–	Self-administered	–	No	Yes	?
AAMQ-13	Mixed method Literature review, Delphi process- expert panel	1	13	1–3	≥30	Self-administered	No	–	Yes	?
MAUQ	Not specified	1	16	–	–	Researcher-administered	–	Yes	No	?
IMAS	Qualitative method: Expert opinion	1	19	–	–	Researcher-administered	Yes	–	Yes	?
ASCD	Not specified	1	8	–	> 29	Self-administered	No	–	Yes	Implementation
ITBQ	Not specified	1	10	–	–	Self-administered	No	–	Yes	?
SMAQ	Not specified	1	6	1.06	–	Self-administered	Yes	–	Yes	?

**Abbreviations: MMAS** -Morisky Medication Adherence Scale, **MGT**-Morisky-Green test, **MAQ**-Medication Adherence Questionnaire, Type 2 **DM**- Type 2 Diabetes Mellitus, **MeDS**-Measure of Drug Self-Management, **DMAS**-Diabetes Medication Adherence Scale, **MAUQ**-Medication Adherence Universal Questionnaire**, MPRAQ**-Medication Practical barriers to Adherence Questionnaire, **MARS**-Medication Adherence Report Scale, **MyMAAT-**Malaysia Medication Adherence Assessment Tool**, HBCTS-**Hill-Bone compliance to high blood pressure therapy scale**, K Wood-MAS-**Krousel-Wood Medication Adherence Scale**, IADMAS-**Iraqi Anti-Diabetic Medication Adherence Scale**, MAT--** Measurement of Adherence to the Treatment**, MCQ-**Medication Compliance Questionnaire**, GMAS-**General Medication Adherence Scale**, AAMQ-**Adherence to Asthma Medication Questionnaire**, ARMS-C-**Adherence to Refills and Medications Scale, Chinese**, TAI-** Test of Adherence to Inhalers**, SEAMS-**Self-Efficacy for Appropriate Medication Use Scale**, MMWFU-** Making Medicines Work For You**, IMAS-** Inhaled Medication Adherence Scale**, MALMAS-**Malaysian Medication Adherence Scale**, TAQPH**- Treatment Adherence Questionnaire for Patient with Hypertension**, PSAM-** Patient Satisfaction with Asthma Medication**, MUAH-**Maastricht Utrecht Adherence in Hypertension**, MASES -** Medication Adherence Self-Efficacy Scale**, SATMED-Q-**Treatment Satisfaction with Medicines, Questionnaire**, ASK-**Adherence Starts with Knowledge**, TSQM-**Treatment Satisfaction Questionnaire for Medication**, BBQ-**Beliefs and Behavior Questionnaire**, LMAS-**Lebanese Medication Adherence Scale**, ChMAR-Scale-**Chinese version of Medication Adherence Reasons Scale**, BMQ-**Beliefs about Medicines Questionnaire**, PDSMS-**Perceived Diabetes Self-Management Scale, **NR**- Not Reported, **HBCS**- Hill Bone Compliance Scale, **M-DRAW-** Modified Drug Adherence Work-up Tool, **MUSE-** Medication Understanding and Use Self Efficacy Scale, **SF**-Short Form, **FATS-** Facilitators of and Barriers to Adherence to Hypertension Treatment Scale, **VAS-** Visual Analogue Scale, **GAS-** General Adherence Scale, **HBMA**- Hill-Bone Medication Adherence scale, **IAQ**- Inhaler Adherence Questionnaire, **SR-** self-report, **HBM-** Health belief model, **ASCD**- Adherence Scale in Chronic Diseases, **ITBQ**- Inhaled Therapy Beliefs Questionnaire, **SMAQ**- Simplified Medication Adherence Questionnaire.

**Key findings**: -

1) Total number of studies = 140 (37 focused on the development of scales and103 (73.6%) focused translating the original scales and then validating them in different settings or languages).

2) The number of questions in these scales ranged between 4–30 questions (Median -11).

3) The time taken to complete answering the scales ranges from 1–40 minutes (Median = 5.53 minutes).

4) Cut-off score for adherence in 22 scales range from 0–1 to 87.

5) Implementation and discontinuation stages of medication taking considered in 8 scales, and implementation alone in 8 scales.

6) Self-efficacy reported in 9 scales.

7) Self-administered - 59.6% of scales, researcher-administered -17.5% and administered by both methods- 22.8%.

8) Only Twenty-eight scales (49%) were validated for patients with low literacy.

The psychometric properties of the included scales were extracted based on key measurement attributes, including reliability (internal consistency, test-retest reliability), validity (content, construct, criterion), sensitivity, specificity, and response rate. These details were derived from the original development and validation studies of each scale. While no single framework was explicitly followed, our approach aligns with established principles of psychometric evaluation [27] to provide a comprehensive synthesis of scale properties. The extracted information is presented in Table 2 and S5 Dataset.

Two different investigators (MJ and PR) verified the data independently for accuracy after extraction. Discrepancy was resolved by discussion with a third investigator (JT). Missing data were addressed systematically by contacting corresponding authors and investigators for full texts and missing data, but no responses were received. Therefore, we included studies that validated medication adherence tools with psychometric properties for analysis. We employed an available data analysis approach, assuming data were missing at random, ensuring reliable and robust results.

### Data synthesis

The data from the included studies were compiled as a descriptive summary. The findings were presented in the form of summary tables based on an initial overview of the general characteristics of the included studies and scales, followed by psychometric properties of scales, and a comparison of medication adherence assessment scales from development and translational validation studies.

### Ethics and dissemination

An ethics review was not required, as only publicly available data was analyzed. Findings from the scoping review will be published in a peer-reviewed journal and disseminated to health professionals and policymakers involved in NCD care.

## Results

### Study selection

**Fig 1:**
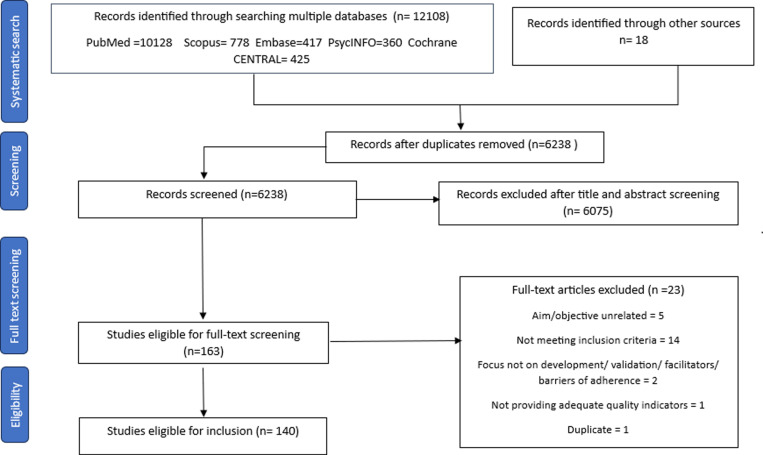
PRISMA flow diagram.

A total of 12108 records were initially identified, which consisted of 6238 articles after removing duplicates. Title and abstract screening excluded 6075 articles, leaving 163 for full-text review (Fig 1: PRISMA flow diagram). A further 23 articles [15,28–42] were excluded for various reasons listed in Table D in S1 Appendix. Ultimately, 140 studies met the inclusion criteria for this review [43–182].

All included studies were appraised using the JBI critical appraisal scales to ensure methodological rigor. Based on established conventions and guidelines [24], cross-sectional studies with scores ≥4 and RCTs with scores ≥6 were considered to be of ‘good quality’ for inclusion. These thresholds were chosen as they represent the minimum level of methodological integrity needed to provide reasonably reliable evidence.

The appraisal revealed that, among cross-sectional studies, 120 scored between 4 and 6 [43–162], while 17 scored between 7 and 8 [163–179]. All three RCTs scored between 8 and 9 (Table B in S1 Appendix) [180–182]. While all included studies met our definition of ‘good quality,’ we acknowledge that there was still some heterogeneity within this range. However, we did not find any significant differences in our review’s overall findings based on whether studies had scores at the higher or lower end of our ‘good quality’ range.

Given that all included studies met our pre-defined quality thresholds, we believe this strengthens the reliability and robustness of our findings. The consistent application of sound methodologies across studies provided a solid foundation for our synthesis and supported the rigor with which we drew our conclusions.

### Study characteristics

A total of 140 studies [43–182], published between January 1950 and June 2024, were analyzed. Among these, 131 studies (93.6%) assessed a single self-report scale [43–51,53–67,70–75,77–91,93–143,145–156,158–165,167–175,177–182], while 9 studies (6.4%) utilized more than one scale [52,68,69,76,92,144,157,166,176]. The sample size ranged from 26 [95] to 6237 [143] with a median of 174.5 participants. Across these studies, fifty-seven individual self-reported adherence scales were identified, with adherence cutoff scores varying from 1 [146] to 87 [150] and non-adherence rates ranging from 0.6% [176] to 89.4% [127].

The most frequently studied scale was the MMAS-8 (n=24, 17.14%) [51,56,59,60,64,69,76,78,81,84,90,97,99,103,106,109,121,126,132,135,168,174,177], followed by the BMQ (n=13, 9.2%) [46,52,54,58,86,88,120,124,133,134,155,157,166], GMAS (n=11, 7.9%) [82,83,101,102,115–118,130,148,175], MMAS-4 (n=9, 6.4%) [92,94,108,112,143–145,166,178], MARS (n=9, 6.4%) [49,52,67,98,105,111,136,138,144] and ARMS (n=7, 5%) [53,77,91,93,107,152,181]. For all the scales, both developmental and translational validation studies were considered. However, the developmental study for the MMAS-8 [183] was excluded from data extraction because it has been retracted.

The MMAS-8 was primarily studied in patients with T2DM (n=12) [51,56,59,69,81,99,103,106,126,135,177] and HTN (n=11) [56,64,76,78,90,97,109,121,132,168,174]. Meanwhile, the GMAS was predominantly used in chronic diseases (n=7) [83,115–117,130,148,175] involving more than 1 select NCD. The detailed study characteristics are summarized in Table 1.

### Psychometric properties of scales with the methods and standards from developmental studies (Table 2)

The scales were developed through a combination of qualitative methodologies (n=4,10.8%) [47,146,147,182], including focus group discussions, semi-structured interviews with patients and experts, as well as an extensive literature review (n=13,35%) [66,67,71,73,85,89,110,112,140,162,165,167,183]. Additionally, mixed methods were employed (n=17,45.95%) in the development process [46,48,61,79,93,114,115,122,123,142,153,154,156,158,161,164].

Response rates were documented in 17 studies (45.95%) [46–48,61,66,67,71,85,110,115,140,146,158,161,162,164,182], ranging from 50.2% [66] to 100% [67]. Sensitivity of scales was assessed in 11 studies (28.9%) [74,79,110,112,114,115,122,146,165,167,169], while specificity was evaluated in 10 studies (26.3%) [74,79,110,112,114,122,146,165,167,169]. Five scales demonstrated high sensitivity [74,110,112,114,167], and one scale exhibited high specificity (80–100%) [114], indicating their efficacy in accurately measuring medication adherence and medication non-adherence. Feasibility was not reported for any of the scales. Heterogeneity in psychometric properties refers to the significant inconsistency in measurement characteristics of a tool (reliability, validity, sensitivity, and specificity) across different medication adherence scales or when the same scale is used in different settings.

#### Reliability.

Cronbach’s alpha was reported by 34 studies (89.5%) [46–48,57,61,65–67,71,74,79,85,89,93,110,112,114,115,122,123,140,142,147,153,154,156,158,161,164,165,179,181,182], with 30 studies demonstrating an acceptable range exceeding 0.7, indicating an excellent level of internal consistency [46–48,57,61,66,67,71,79,85,89,93,110,114,115,122,123,140,142,147,153,154,156,158,161,164,179,181,182]. Test-retest reliability, indicating the stability of measurements over time, was reported using Pearson’s or Spearman’s correlation coefficient. Three scales demonstrated high reliability (>0.90) [57,67,115], one scale exhibited moderate reliability (0.8–0.9) [110], and nine scales showed insufficient reliability (<0.8) [66,74,79,85,93,114,123,142,165]. Other reliability measures are summarized in Table 2.

#### Validity.

Ten scales (26.3%) [61,67,74,110,112,122,123,156,179,182] were assessed for their correlation with an objective measure of adherence, with one scale evaluated using MEMS [182], while the others were compared against clinical outcomes such as blood pressure [61,67,112,156] and HbA1c levels [74,110,179]. Criterion-related validity was examined for 56.8% (n=21/37) of the scales [46,47,61,67,71,74,85,93,110,112,114,115,122,123,153,154,156,158,165,167,179], revealing significant correlations with various measures including clinical outcomes [61,67,74,110,112,123,156,179], electronic adherence measurements [122,182], subscales of the current scale [46,93,115,154], and other self-report scales [47,61,65–67,74,79,85,93,110,114,122,123,142,146,153,158,164,167,169,182]. Construct validity, a pivotal aspect that assess whether a scale accurately measures the theoretical construct, it is intended to evaluate- which was undertaken for 73% (n=27) of the scales [46–48,61,65–67,74,79,89,110,112,114,115,122,140,142,146,153,154,158,161,164,165,181,182].

On reviewing psychometric properties of translational studies, 6 scales showed high sensitivity [49,100,109,113,116,133], while 11 demonstrated high specificity (80–100%) [56,76,80,113,116,133,137,143,144,150,152]. Sixty six studies (47.14%) reported good internal consistency with Cronbach’s alpha >0.7 [45,49,52–56,58,59,62,63,68–70,75,77,78,80–82,86–88,91,92,95–97,99,101,102,104,107,111,113,116–120,124,125,127–132,134,137–139,148,149,151,152,155,157,159,160,163,166,171,173,175,180], whereas only 3 studies demonstrated high reliability (>0.90) [102,109,128]. Seventy-four studies (52.9%) utilized a correlation with a comparative measure. The response rates in these studies varied between 24.7% [150] and 99% [96]. Among these, 37 studies (26.4%) compared adherence against objective measures such as pill count, the MedSignals pillbox, and HbA1c levels, while the remaining studies were against self-report questionnaires. Other measures are summarized in Table E in S1 Appendix.

### Medication adherence assessment scale comparison

Out of the 140 studies in total, 37 focused specifically on the development of scales [46–48,57,61,65–67,71,74,79,85,89,93,110,112,114,115,122,123,140,142,146,147,153,154,156,158,161,162,164,165,167,169,179,181,182], whereas 103 (73.6%) were primarily concerned with translating the original scales and then validating them in different settings or languages [43–45,49–56,58–60,62–64,68–70,72,73,75–78,80–84,86–88,90–92,94–109,111,113,116–121,124–139,141,143–145,148–152,155,157,159,160,163,166,168,170–178,180].

The number of questions in these scales varied from 4 [112] to 30 [46] questions, with a median count of 11 questions. The time taken to complete answering the scales varied from 1 minute [137] to approximately 40 minutes [132], with a median duration of 5.53 minutes. A scale of 13 questions took a minimum of 1–3 minutes [114], while 40 minutes were required to answer the scale with 8 questions [132]. Regarding adherence, 22 scales specified a cut-off score, with the reported range varying from 0–1 [146] to 87 [150]. Implementation and discontinuation stages of medication taking were considered in 8 scales [48,49,67,93,112,142,160,181], whereas, only the implementation stage was considered in 8 scales [45,48,70,79,87,89,180,182].

Self-efficacy has been found to be a crucial predictor of adherence, with 9 scales reported for its assessment [46,65,70,89,123,156,161,162,169]. Notably, SEAMS [123], TSQM [161], and MASES [156] were specifically developed to integrate self-efficacy into the measurement of adherence. The majority of the scales, 59.6%, were self-administered, while researcher-administered scales, conducted in consultation with patients, followed closely at 17.5%. Only 22.8% of scales were administered by both methods. Only Twenty-eight scales (49%) were validated for patients with low literacy [44,51,52,57,61,69–71,74,79,87,93,108,110,115,122,124,129,139,140,146,147,150,158,163,165,167], making them suitable for use across all literacy levels (Table 3).

Forty-two (73.7%) self-report scales were developed to assess medication adherence among multiple conditions [44–48,51,61,65–71,74,76,79,85,93,111,112,115,123,128,131,140,142,146,150,156–158,160–163,167,169,171,176,180], whereas 15 (26.3%) were tailored to specific conditions [43,57,62,69,72,75,110,113,114,137,147,154,164,165,182]. These scales evaluated adherence to particular medications such as antihypertensives [89] or inhalers [147]. Some either created new tools, like the Diabetes Medication Adherence Scale (DMAS) [165] or adopted established validated measures, like the Hill-Bone Medication Adherence scale (HBMA) [131].

Among these, 4 scales were designed for assessing medication adherence in HTN [62,75,89,182]. with the HBCTS [89] being the most commonly utilized. For T2DM, 4 scales were identified [57,69,110,165], while 3 were tailored for BA [114,154,164], and 4 others focused on inhalational medicines [43,113,137,147] applicable to both BA and COPD. Conversely, non-condition-specific measures evaluated adherence more broadly [44–48,51,61,65–71,74,76,79,85,93,111,112,115,123,128,131,140,142,146,150,156–158,160–163,167,169,171,176,180], with most studies employing pre-existing validated questionnaires, such as MMAS-8 and ARMS.

### Domains and gaps of existing scales

Among the 57 individual medication adherence assessment scales identified, most of the scales evaluated medication-taking behavior, while only a few did not assess this domain [43,46,70,123,154,156–158,161,162]. The BMQ [46], HBM-based questionnaire [157], and ITBQ [43] concentrate on beliefs, concerns, and perceptions. Whereas, the TSQM [161], SATMED-Q [158], and PSAM [154] focus on domains such as convenience, satisfaction, and effectiveness. SEAMS [123] and MASES [156] address patients’ self-efficacy, motivation, and confidence.

Although some scales, like the GMAS [115], LMAS [167], MAR-Scale [142], and DMAS [165], consider the cost factor, they do not adequately address the out-of-pocket expenditure, which is an important area of health economics especially in developing countries where most patients pay direct medical costs which is a crucial aspect of health economics in developing countries where most patients bear direct medical costs.

The best known and most widely used is the Morisky Scale developed from the 4-item MMAS-4 [112] to the 8-item MMAS-8 [183]. The scale identifies barriers such as forgetfulness and adverse effects and fails to capture cost-related non-adherence (CRNA), self-efficacy, and health care system-related factors. In addition, there is overlap between the questions which could result in scoring ambiguity.

The MARS [49] explores beliefs and barriers to medication-taking behavior. It includes ten questions that assess adherence behavior and disease control over the past week and used for patients with chronic mental illness. The major limitations were the scale not validated in low literacy patients and did not assess self-efficacy.

The ARMS [93], evaluates taking medications as prescribed and refilling medications on schedule. Cost factor and out of pocket expenditure, and patient self-efficacy dimensions of medication adherence were not considered in this scale. Additionally, sensitivity, specificity of ARMS are not reported and compared to clinical outcomes. We do not find any studies that have validated ARMS in the Indian setting.

The SEAMS [123] and the BMQ [46] have three main question headings and multiple sub questions. Both assess self-efficacy, barriers and are validated in patients with low literacy. However, the sensitivity and specificity of SEAMS is not estimated, and it lacks the ability to rapidly estimate adherence at point of care/bedside. Additional information on the domains and gaps of scales are provided in Table 4.

**Table 4:** Domains and gaps of medication adherence assessment scales.

Scale name	Domains assessed	Strengths	Limitations	Cut-off score
MMAS 8	Patient, Therapy, Condition Factors	Validated in low literacy Stage of medication taking: Implementation, discontinuation	No cost assessment Overlapping questions	>6
BMQ	Beliefs, concerns, and Self-efficacy	Captures patient beliefs Validated in low literacy	No cost assessment Lacks rapid bedside assessment Stage of medication taking unidentified	–
GMAS	Patient, Therapy, Condition and Cost Factors	Validated in low literacy Includes cost assessment	Lacks self-efficacy No social support assessment Stage of medication taking unidentified	≥ 27
MMAS 4	Patient, Therapy, Condition Factors	Stage of medication taking: Implementation, discontinuation	No cost assessment Overlapping questions License agreement of MMAS Not validated in low literacy patients	<4
MARS-5	Patient-related	Validated in low literacy Simple scoring Stage of medication taking: Implementation, discontinuation	No cost assessment No therapy/condition factors	≥20
ARMS	Patient, Therapy, condition and Refill Factors	Includes refill adherence Validated in low literacy Stage of medication taking: Implementation, discontinuation	No social support assessment Limited data on sensitivity	12–20
HBCTS	Patient, Therapy, condition, diet and Refill Factors	Self-efficacy assessed Validated in low literacy patients Stage of medication taking: Implementation	Single disease, Limited in generalizability No cost assessment Limited generalizability	–
MARS-10	Patient, Therapy, Condition Factors	Stage of medication taking: Implementation, discontinuation	Not validated in low literacy patients Limited data on sensitivity	≥ 4.5
TSQM Version I and II	Satisfaction on treatment effectiveness, Convenience of therapy	Domain characteristics	Stage of medication taking not identified Not validated in low literacy patients Lacks rapid bedside assessment No patient behavior/self-efficacy No cost assessment	–
LMAS	Patient, Therapy, Condition, Cost, Psychological, Refill factors	Validated in low literacy	Stage of medication taking not identified	38
SEAMS	Self-efficacy or confidence	Validated in low literacy	No cost assessment Limited data on sensitivity Stage of medication taking not identified Lacks rapid bedside assessment	–
M-DRAW	Patient, Therapy, Condition, Health care system and social related factor	Domain characteristics	No cost assessment Stage of medication taking not identified Not validated in low literacy patients	–
MAR-Scale	Patient, Therapy, Cost related and psychological factor	Domain characteristics	No cost assessment Stage of medication taking not identified Not validated in low literacy patients	>15
SPUR – 27	Patient, Therapy, Condition, Health care system, Belief, Perceptions and Motivation related factors	Validated in low literacy	No cost assessment Stage of medication taking not identified	87
TAQPH	Patient, Therapy, Condition, Healthy diet and lifestyle factors	Domain characteristics	Single disease, Limited generalizability No cost assessment Not validated in low literacy patients Stage of medication taking not identified	–
MASES-SF	Self-efficacy	Validated in low literacy Stage of medication taking: Implementation	No cost assessment Lacks rapid bedside assessment	–
MCQ	Patient, Therapy and Condition factors	Validated in low literacy	No cost assessment Stage of medication taking not identified No self-efficacy/social support assessment	≥ 27
A 14-item scale	Patient, Therapy, Condition, Cost, Refill and Psychological related factor	Domain characteristics	Stage of medication taking not identified Not validated in low literacy patients Overlapping questions No self-efficacy/social support	50–56
A 12-item Scale	Patient, Therapy, Healthcare, Awareness, Motivation	Validated in low literacy	No cost assessment Stage of medication taking not identified No socio-economic/ self-efficacy assessment	–
DMAS	Patient, Therapy, Condition, Cost, Cultural and Psychological related factor	Validated in low literacy	Single disease, Limited in generalizability Stage of medication taking not identified No self-efficacy assessment	7
TAI questionnaire	Patient, Therapy, Condition, Cost, Occupational and Psychological related factor	Validated in low literacy	Single dosage form, Limited in generalizability Overlapping questions No self-efficacy assessment Stage of medication taking not identified	50
MASES	Self-efficacy	Validated in low literacy Stage of medication taking: Implementation	No cost assessment Lacks rapid bedside assessment	–
HBMA scale	Patient, Condition and Refill related factor	Domain characteristics	No socio-economic/ self-efficacy assessment, No cost assessment Not validated in low literacy patients Stage of medication taking not identified	–
IAQ	Patient, Condition and Psychological related domain	Domain characteristics	Single dosage form, Limited in generalizability No socio-economic/ self-efficacy assessment, No cost assessment Stage of medication taking not identified Not validated in low literacy patients	–
SR-4scale	Patient and Therapy related factors	Domain characteristics	No socio-economic/ self-efficacy assessment, No cost assessment Stage of medication taking not identified Not validated in low literacy patients	>2
HBM -based questionnaire	Patient, Condition, Health system, Motivation **and** Belief related factors	Domain characteristics	No cost assessment Lacks rapid bedside assessment Stage of medication taking not identified Not validated in low literacy patients	–
ASK-20	Patient, Therapy, Condition, Health system, cost, Psychological, Refill related factors	Stage of medication taking: Implementation, discontinuation	No socio-economic/ self-efficacy assessment Not validated in low literacy patients	–
ASK -12	Patient, Therapy, Condition, Health system, cost, Psychological, Refill related factors	Stage of medication taking: Implementation, discontinuation	No socio-economic/ self-efficacy assessment Not validated in low literacy patients	–
PSAM	Satisfaction, Perception and Trust in treatment	Validated in low literacy	Single disease, Limited in generalizability No cost assessment Lacks rapid bedside assessment Stage of medication taking not identified	–
BBQ	Patient, Therapy, Health system, Cost, Confidence and satisfaction related factor	Domain characteristics	No socio-economic/ self-efficacy assessment, No cost assessment Stage of medication taking not identified Not validated in low literacy patients	25
MUAH	Patient, Therapy, Condition, Health system, Positive attitude, social support, Diet, exercise and lifestyle related factors	Stage of medication taking: Implementation	No self-efficacy assessment No cost assessment Not validated in low literacy patients	–
SATMED-Q	Treatment effectiveness, Convenience and Global satisfaction	Domain characteristics	No cost assessment Lacks rapid bedside assessment No self-efficacy assessment Stage of medication taking not identified Not validated in low literacy patients	–
K Wood-MAS-4	Patient related and Self-efficacy factors	Domain characteristics	No socio-economic assessment No cost assessment Stage of medication taking not identified Not validated in low literacy patients	–
MeDS	Patient, Therapy, Health system, Cost, Psychological and motivation related factors	Validated in low literacy	Overlapping questions Stage of medication taking not identified	–
MAQ for Asthma	Patient, Therapy, Condition, Psychological, Patient’s attitude, Perception and belief related factors	Domain characteristics	Single disease, Limited in generalizability No socio-economic/ self-efficacy assessment, No cost assessment Stage of medication taking not identified Not validated in low literacy	–
MAQ for DM	Patient, Therapy, Condition and diet related factors	Validated in low literacy	Single disease, Limited in generalizability No socio-economic/ self-efficacy assessment, No cost assessment Stage of medication taking not identified	–
IADMAS	Patient, Therapy, Cost and Psychological factors	Validated in low literacy	Single disease, Limited in generalizability No socio-economic/ self-efficacy assessment, No cost assessment Stage of medication taking not identified	8
MMWFU	Patient, Therapy, Cost and Psychological factors	Domain characteristics	No socio-economic/ self-efficacy assessment, No cost assessment Stage of medication taking not identified Not validated in low literacy	0-1
ChMAR-Scale	Patient, Therapy, Condition, Cost, Perception, Access and Psychological factors	Validated in low literacy	No socio-economic/ self-efficacy assessment, No cost assessment Stage of medication taking not identified	–
MyMAAT-12	Patient, Therapy, Perceived barriers, utility, Socio-cognitive, Refill and Psychological factors	Validated in low literacy Stage of medication taking: Implementation	No cost assessment	> 54
MPRAQ	Patient, Therapy, Cost and Refill related factors	Domain characteristics	Overlapping questions Not validated in low literacy Stage of medication taking not identified	–
AAMQ-13	Patient, Therapy, Condition, Health care system, Cost, Belief and Psychological factor	Domain characteristics	Single disease, Limited in generalizability No socio-economic/ self-efficacy assessment Not validated in low literacy Stage of medication taking not identified	≥ 30
MAUQ	Patient, Therapy, Condition, Self-efficacy, Exercise, diet and lifestyle related factors	Domain characteristics	Overlapping questions Stage of medication taking not identified No cost assessment Not validated in low literacy	–
ASCD	Patient, Therapy, Condition and cost related factors	Stage of medication taking: Implementation	No socio-economic/ self-efficacy assessment Not validated in low literacy	≥ 29
ITBQ	Beliefs and Perceptions related factors	Domain characteristics	No cost assessment Lacks rapid bedside assessment Single dosage form, Limited in generalizability Self-efficacy not assessed Stage of medication taking not identified Not validated in low literacy	–
SMAQ	Patient, Therapy and Psychological related factors	Validated in low literacy	Overlapping questions No socio-economic/ self-efficacy assessment, No cost assessment Stage of medication taking not identified	–

**Abbreviations: MMAS** -Morisky Medication Adherence Scale, **BMQ-**Beliefs about Medicines Questionnaire**, GMAS**- General Medication Adherence Scale, **MGT**-Morisky-Green test, **MAQ**-Medication Adherence Questionnaire, **T2DM**-Type 2 Diabetes Mellitus, **MeDS**-Measure of Drug Self-Management, **DMAS**-Diabetes Medication Adherence Scale, **MAUQ**-Medication Adherence Universal Questionnaire**, MPRAQ**-Medication Practical barriers to Adherence Questionnaire, **MARS**-Medication Adherence Report Scale, **MyMAAT-**Malaysia Medication Adherence Assessment Tool**, HBCTS-**Hill-Bone compliance to high blood pressure therapy scale**, K Wood-MAS-**Krousel-Wood Medication Adherence Scale**, IADMAS-**Iraqi Anti-Diabetic Medication Adherence Scale**, ARMS** - Adherence to Refills and Medications Scale, **SEAMS** - Self-Efficacy for Appropriate Medication Use Scale, **LMAS-**Lebanese Medication Adherence Scale**, ChMAR-Scale-**Chinese version of Medication Adherence Reasons Scale**, BBQ-**Beliefs and Behavior Questionnaire**, SATMED-Q-**Treatment Satisfaction with Medicines, Questionnaire**, ASK-**Adherence Starts with Knowledge**, TSQM-**Treatment Satisfaction Questionnaire for Medication**, MUAH-**Maastricht Utrecht Adherence in Hypertension**, MASES -** Medication Adherence Self-Efficacy Scale**, MCQ-**Medication Compliance Questionnaire**, TAQPH**- Treatment Adherence Questionnaire for Patient with Hypertension**, MCQ-**Medication Compliance Questionnaire**, PSAM-** Patient Satisfaction with Asthma Medication**, TAI-** Test of Adherence to Inhalers**, HBCS** – Hill Bone compliance scale, **MASES-SF**- Medication Adherence Self-Efficacy Scale-Short Form, **HBMA**- Hill-Bone Medication Adherence scale, **IAQ**- Inhaler Adherence Questionnaire, **MMWFU-** Making Medicines Work For You, **AAMQ-** Adherence to Asthma Medication Questionnaire, **M-DRAW-** Modified Drug Adherence Work-up Tool, **M-DRAW-** Modified Drug Adherence Work-up Tool, **SR-** self-report, **HBM-** Health belief model, **WHO-**World Health Organization, **ASCD**- Adherence Scale in Chronic Diseases, **ITBQ**- Inhaled Therapy Beliefs Questionnaire, **SMAQ**- Simplified Medication Adherence Questionnaire.

## Discussion

This scoping review synthesizes evidence on medication adherence scales for NCDs, identifying critical gaps in their design, validation, and applicability. While many scales, such as MMAS, GMAS, and BMQ, are widely used, they often fail to capture the multifaceted nature of adherence, particularly in populations with low literacy and multimorbidity. Developing countries facing an epidemiological transition are witnessing a rising burden of multiple NCDs. Given the prevalence of comorbidities among patients managing multiple medications, an adherence scale tailored for multiple NCDs is essential for accurate evaluation and targeted interventions

Most existing tools focus on self-reported adherence, which is prone to recall and social desirability biases, reducing their reliability in real-world settings [67,112]. Integrating objective methods such as electronic pill monitoring or biochemical markers alongside self-reports can mitigate these challenges.

A major limitation in current adherence measurement is the lack of consideration for socioeconomic and cultural factors. Many scales focus predominantly on behavioral adherence while neglecting critical influences such as financial constraints, access to medications, cultural beliefs, and healthcare system challenges, which are particularly significant in low- and middle-income countries (LMICs). These challenges shape patients’ perceptions of medication necessity and efficacy [184]. Economic factors, including out-of-pocket expenditures and access to healthcare services, play a significant role in medication adherence, especially in developing countries like India [185]. Indirect healthcare costs, including transportation expenses, can lead to missed clinic appointments and reduced access to pharmacies [186,187]. Overlooking these aspects may lead to inaccurate adherence assessments and ineffective interventions [188]. The inclusion of constructs related to financial burden, treatment-related stress, and patient-provider communication could improve the contextual relevance of adherence assessments in LMICs [189,190].

Without robust cross-cultural validation, existing adherence scales may not generalize to diverse healthcare settings, increasing the risk of measurement bias and limiting their utility in resource-limited contexts. Tools like the GMAS require broader adaptation to enhance their relevance beyond specific regional settings. Additionally, validating these scales across different healthcare contexts ensures their generalizability beyond hospital-based and urban cohorts [22]. Expanding their application can enhance real-world usability and increase their adoption in healthcare systems with varied socioeconomic backgrounds.

From a clinical perspective, adherence scales should incorporate factors such as patient engagement, shared decision-making, and trust in healthcare providers, as these elements significantly impact medication-taking behavior [62]. As evidenced in a study by Gellad W F et al. [7], hypertensive patients who discussed their condition with their physician were significantly more likely to be adherent to their blood pressure medication.

Despite their critical role in adherence, current scales lack structured assessments of trust and shared decision-making [191], limiting their applicability in patient-centered care. Incorporating these dimensions into adherence assessment tools could improve their predictive validity and real-world applicability. In addition, the use of patient-centered communication strategies, such as motivational interviewing, has shown promise in improving adherence and should be explored in future scale development [192].

Psychosocial factors, including stress and mental health conditions, significantly influence medication adherence. A study by Kretchy I A et al. [193] found that individuals experiencing stress were significantly more likely to exhibit nonadherence compared to those with low or no stress levels. Patients displaying stress symptoms might be more vulnerable to the adverse effects of their medications, often leading to discontinuation [193]. The psychological dimensions of medication adherence have been considered in the Lebanese Medication Adherence Scale [167]. However, these psychological factors were not incorporated into most other scales we reviewed. Evolving medication adherence scales should explicitly integrate these dimensions to ensure a comprehensive and accurate assessment. Emerging evidence also suggests that integrating patient-reported outcome measures (PROMs) related to mental health into adherence scales can improve predictive validity [194].

Methodologically, the use of qualitative inputs from patients and experts during item development, as seen in SEAMS and GMAS, can guide the creation of more nuanced tools. The exploratory sequential mixed-method approach, involving qualitative and quantitative components, is widely recognized for scale construct development and validation [195]. While internal consistency is frequently reported (e.g., Cronbach’s alpha > 0.7 for most scales), fewer studies assess test-retest reliability or sensitivity and specificity. Analysis of the psychometric properties of various scales revealed significant variability in sensitivity, specificity, reliability, and validity across different settings. For instance, the MMAS-8 showed varying sensitivity across different studies [51,56,59,76,126], which may not be universally applicable. Future scale development must employ robust psychometric analyses to ensure temporal stability and diagnostic accuracy. Scales such as the MMAS-4 and BMQ have shown effectiveness but require contextual validation in low-literacy populations and under-resourced healthcare settings. Developing pictorial or voice-assisted tools could improve accessibility and usability in these populations.

Current scales, like MMAS-8, predominantly assess implementation. A meta-analysis by DiMatteo et al. reported that about 24% of patients discontinue their medication prematurely, which can significantly impact treatment outcomes [196]. Future tools should evaluate all stages of adherence—initiation, implementation, and discontinuation—to provide a comprehensive understanding of medication-taking behaviors. Given the limitations of self-reported adherence, integrating digital health solutions—such as mobile-based adherence interventions and electronic monitoring—can provide real-time insights and reduce recall bias. These technologies can complement traditional adherence scales, enhancing both measurement accuracy and patient engagement [197].

Bridging these gaps through participatory research and rigorous validation will yield adherence assessment tools that are not only reliable and inclusive but also aligned with real-world patient needs. Strengthening adherence measurement methodologies will not only improve clinical outcomes but also optimize healthcare resource utilization globally.

This review was limited to English-language studies for feasibility reasons, which may have led to the exclusion of relevant adherence scales published in other languages. This linguistic restriction is a methodological limitation, potentially impacting the comprehensiveness of our findings, particularly in non-English-speaking regions.

## Conclusion

In conclusion, this scoping review emphasizes the complexity of measuring medication adherence for NCDs through scales like MMAS-8, GMAS, and BMQ. While these tools are valuable, they fail to capture key factors such as socio-economic, cultural influences, and multimorbidity—especially in low-literacy populations. Most scales address only parts of the medication-taking process and lack rigorous psychometric validation using objective measures like Medication Event Monitoring System (MEMS). In light of these findings, it is clear that the existing adherence scales should not only be revised but also rethought to address the evolving challenges in patient care. These updated scales must be developed using a comprehensive, evidence-based approach, considering the dynamic nature of patient behaviors and the complex context in which they exist.

Future scale development should focus on creating context-specific, culturally sensitive tools that assess adherence in patients with multiple NCDs. These tools should incorporate factors such as patient trust, stress, and beliefs and employ a robust methodological framework with advanced algorithms which can improve the predictive power and reliability of these tools. Such improvements could ultimately lead to more precise adherence measurement and better patient outcomes, enhancing both clinical practice and research efforts.

## Supporting information

S1 Appendix Search Strategy, Critical appraisal of articles, Scoring developed for JBI critical appraisal checklist, List of excluded studies and reasons for exclusion and Psychometric properties of translational studies of scales. (PDF)

S2 Dataset Data charting excel and data extracted from studies. (XLSX)

S3 Text PRISMA-ScR-Checklist. (DOCX)

S4 Text Scoping review protocol. (PDF)

S5 Dataset Study screening and selection process. (XLSX)
