# Supplementary material for: Medication adherence scales in non-communicable diseases: A scoping review of design gaps, constructs and validation processes
Source: PLoS One. 2025 May 14;20(5):e0321423. doi: 10.1371/journal.pone.0321423 (PMC12077792; doi:10.1371/journal.pone.0321423)
Supplement: S1 Appendix — (PDF) [file pone.0321423.s001.pdf]

S1 Appendix

Decoding medication adherence scales in Non Communicable Diseases- A Scoping Review

Contents

Table A: Search Strategy....3
Table B: Critical appraisal of articles....11
Table C: Scoring developed for JBI critical appraisal checklist....17
Table D: List of excluded studies and reasons for exclusion....18
Table E: Psychometric properties of translational studies of scales....19
REFERENCES....21

Table A: Search Strategy

Resource: PUBMED

Date of search: 15/11/2023

Sl.no.	Search	No. of articles	Filter
#1	((((((((“medication adherence*”[Title/Abstract]) OR (“medication compliance*”[Title/Abstract]) OR (“medication non adherence*”[Title/Abstract]) OR (“medication noncompliance*”[Title/Abstract]) OR (“medication persistence*”[Title/Abstract]) OR (“Medication Adherence”[Mesh])) OR (“Drug adherence*”[Title/Abstract])) OR (“Drug compliance*”[Title/Abstract])) OR (“Drug noncompliance*”[Title/Abstract])) OR (“drug non adherence[Title/Abstract])	37,477	1950/1/1-2023/11/10
#2	((((((((((((((((Survey*[Title/Abstract]) OR (“Questionnaire development*”[Title/Abstract])) OR (Scale*[Title/Abstract])) OR (“Morisky scale*”[Title/Abstract])) OR (Morisky*[Title/Abstract])) OR (MMAS*[Title/Abstract])) OR (“Hill Bone*”[Title/Abstract])) OR (MARS*[Title/Abstract])) OR (“Medication Adherence Report Scale*”[Title/Abstract])) OR (ARMS*[Title/Abstract])) OR (“Brief Medication Questionnaire*”[Title/Abstract])) OR (“Compliance scale*” [Title/Abstract])) OR (“Adherence scale*”[Title/Abstract])) OR (“Medication Questionnaire*”[Title/Abstract])) OR (“Self-Efficacy for Appropriate Medication Use Scale*”[Title/Abstract])) OR (“Medication Use Scale*”[Title/Abstract])) OR (“Adherence Report Scale*”[Title/Abstract])) OR (“Surveys and Questionnaires”[Mesh])) OR (“Self-Report”[Mesh]))	2,812,385	1950/1/1-2023/11/10
#3	((((((((((((((((((((("Non-communicable diseases*"[Title/Abstract]) OR ("non-infectious diseases*"[Title/Abstract])) OR ("Non-communicable Chronic Diseases*"[Title/Abstract])) OR ("Noninfectious Diseases*"[Title/Abstract])) OR ("Noncommunicable Diseases"[Mesh])) OR ("Type 2 diabetes mellitus*"[Title/Abstract])) OR ("Type 2 diabetes*"[Title/Abstract])) OR ("Type 2 DM*"[Title/Abstract])) OR ("Diabetes*"[Title/Abstract])) OR ("Diabetes Mellitus, Type 2"[Mesh])) OR (Hypertension*[Title/Abstract])) OR (systemic hypertension*[Title/Abstract])) OR ("Hypertension"[Mesh])) OR (Asthma*[Title/Abstract])) OR ("Bronchial asthma*"[Title/Abstract])) OR ("Asthma"[Mesh])) OR (COPD*[Title/Abstract])) OR ("Chronic obstructive pulmonary disease*"[Title/Abstract])) OR ("Pulmonary Disease, Chronic Obstructive"[Mesh])) OR (CAD[Title/Abstract])) OR ("Coronary artery disease*"[Title/Abstract])) OR ("ischemic heart disease*"[Title/Abstract])) OR ("Stable angina*"[Title/Abstract])) OR ("Coronary Artery Disease"[Mesh])) OR ("Angina, Stable"[Mesh])	1,585,633	1950/1/1-2023/11/10
#4	((((validation*[Title/Abstract]) OR (Validity*[Title/Abstract])) OR (development*[Title/Abstract])) OR (qualitative*[Title/Abstract])) OR ("Reproducibility of Results"[Mesh])	4,103,187	1950/1/1-2023/11/10
#5	#1 AND #2 AND #3 AND #4	480	1950/1/1-2023/11/10

#6	#1 AND #2 AND #3	2883	1950/1/1-2023/11/10
#7	#1 AND #2 AND #3 OR #4	4,105,590	1950/1/1-2023/11/10
#8	#1 AND #2 AND #4	1929	1950/1/1-2023/11/10
#9	#1 AND #2 AND #4 OR #3	1,587,171	1950/1/1-2023/11/10
#10	((("Morisky*"[Title/Abstract]) OR ("Morisky scale*"[Title/Abstract])) OR (MMAS*[Title/Abstract]) AND (2020/1/1:2022/12/31)) AND (((((((((((((((((((((((("Non-communicable diseases*"[Title/Abstract]) OR ("non-infectious diseases*"[Title/Abstract])) OR ("Non-communicable Chronic Diseases*"[Title/Abstract])) OR ("Noninfectious Diseases*"[Title/Abstract])) OR ("Noncommunicable Diseases"[Mesh])) OR ("Type 2 diabetes mellitus*"[Title/Abstract])) OR ("Type 2 diabetes*"[Title/Abstract])) OR ("Type 2 DM*"[Title/Abstract])) OR ("Diabetes*"[Title/Abstract])) OR ("Diabetes Mellitus, Type 2"[Mesh])) OR (Hypertension*[Title/Abstract])) OR (systemic hypertension*[Title/Abstract])) OR ("Hypertension"[Mesh])) OR (Asthma*[Title/Abstract])) OR ("Bronchial asthma*"[Title/Abstract])) OR ("Asthma"[Mesh])) OR (COPD*[Title/Abstract])) OR ("Chronic obstructive pulmonary disease*"[Title/Abstract])) OR ("Pulmonary Disease, Chronic Obstructive"[Mesh])) OR (CAD[Title/Abstract])) OR ("Coronary artery disease*"[Title/Abstract])) OR ("ischemic heart disease*"[Title/Abstract])) OR ("Stable angina*"[Title/Abstract])) OR ("Coronary Artery Disease"[Mesh])) OR ("Angina, Stable"[Mesh]) AND (1950/1/1:2023/11/10))	166	1950/1/1-2023/11/10
#11	((MARS*[Title/Abstract]) OR ("Medication Adherence Report Scale*"[Title/Abstract]) AND (2020/1/1:2022/12/31)) AND (((((((((((((((((((((((("Non-communicable diseases*"[Title/Abstract]) OR ("non-infectious diseases*"[Title/Abstract])) OR ("Non-communicable Chronic Diseases*"[Title/Abstract])) OR ("Noninfectious Diseases*"[Title/Abstract])) OR ("Noncommunicable Diseases"[Mesh])) OR ("Type 2 diabetes mellitus*"[Title/Abstract])) OR ("Type 2 diabetes*"[Title/Abstract])) OR ("Type 2 DM*"[Title/Abstract])) OR ("Diabetes*"[Title/Abstract])) OR ("Diabetes Mellitus, Type 2"[Mesh])) OR (Hypertension*[Title/Abstract])) OR (systemic hypertension*[Title/Abstract])) OR ("Hypertension"[Mesh])) OR (Asthma*[Title/Abstract])) OR ("Bronchial asthma*"[Title/Abstract])) OR ("Asthma"[Mesh])) OR (COPD*[Title/Abstract])) OR ("Chronic obstructive pulmonary disease*"[Title/Abstract])) OR ("Pulmonary Disease, Chronic Obstructive"[Mesh])) OR (CAD[Title/Abstract])) OR ("Coronary artery disease*"[Title/Abstract])) OR ("ischemic heart disease*"[Title/Abstract])) OR ("Stable angina*"[Title/Abstract])) OR ("Coronary Artery Disease"[Mesh])) OR ("Angina, Stable"[Mesh]) AND (1950/1/1:2023/11/10))	166	1950/1/1-2023/11/10
#12	(("Hill Bone*"[Title/Abstract]) OR ("Hill Bone scale*"[Title/Abstract]) AND (2020/1/1:2022/12/31)) AND (((((((((((((((((((((((("Non-communicable diseases*"[Title/Abstract]) OR ("non-infectious diseases*"[Title/Abstract])) OR ("Non-communicable Chronic Diseases*"[Title/Abstract])) OR ("Noninfectious Diseases*"[Title/Abstract])) OR ("Noncommunicable Diseases"[Mesh])) OR ("Type 2 diabetes mellitus*"[Title/Abstract])) OR ("Type 2 diabetes*"[Title/Abstract])) OR ("Type 2 DM*"[Title/Abstract])) OR ("Diabetes*"[Title/Abstract])) OR ("Diabetes Mellitus, Type 2"[Mesh])) OR (Hypertension*[Title/Abstract])) OR (systemic hypertension*[Title/Abstract])) OR ("Hypertension"[Mesh])) OR (Asthma*[Title/Abstract])) OR ("Bronchial asthma*"[Title/Abstract])) OR	22	1950/1/1-2023/11/10

	("Asthma"[Mesh])) OR (COPD*[Title/Abstract])) OR ("Chronic obstructive pulmonary disease*"[Title/Abstract])) OR ("Pulmonary Disease, Chronic Obstructive"[Mesh])) OR (CAD[Title/Abstract])) OR ("Coronary artery disease*"[Title/Abstract])) OR ("ischemic heart disease*"[Title/Abstract])) OR ("Stable angina*"[Title/Abstract])) OR ("Coronary Artery Disease"[Mesh])) OR ("Angina, Stable"[Mesh])) AND (1950/1/1:2023/11/10))		
#13	((ARMS*[Title/Abstract]) OR ("Adherence to Refills and Medications Scale*"[Title/Abstract]))) AND ((((((((((((((((((((((((("Non-communicable diseases*"[Title/Abstract]) OR ("non-infectious diseases*"[Title/Abstract])) OR ("Non-communicable Chronic Diseases*"[Title/Abstract])) OR ("Noninfectious Diseases*"[Title/Abstract])) OR ("Noncommunicable Diseases"[Mesh])) OR ("Type 2 diabetes mellitus*"[Title/Abstract])) OR ("Type 2 diabetes*"[Title/Abstract])) OR ("Type 2 DM*"[Title/Abstract])) OR ("Diabetes*"[Title/Abstract])) OR ("Diabetes Mellitus, Type 2"[Mesh])) OR (Hypertension*[Title/Abstract])) OR (systemic hypertension*[Title/Abstract])) OR ("Hypertension"[Mesh])) OR (Asthma*[Title/Abstract])) OR ("Bronchial asthma*"[Title/Abstract])) OR ("Asthma"[Mesh])) OR (COPD*[Title/Abstract])) OR ("Chronic obstructive pulmonary disease*"[Title/Abstract])) OR ("Pulmonary Disease, Chronic Obstructive"[Mesh])) OR (CAD[Title/Abstract])) OR ("Coronary artery disease*"[Title/Abstract])) OR ("ischemic heart disease*"[Title/Abstract])) OR ("Stable angina*"[Title/Abstract])) OR ("Coronary Artery Disease"[Mesh])) OR ("Angina, Stable"[Mesh])))	1045	1950/1/1-2023/11/10
#14	("Brief Medication Questionnaire*"[Title/Abstract])) AND ((((((((((((((((((((((((("Non-communicable diseases*"[Title/Abstract]) OR ("non-infectious diseases*"[Title/Abstract])) OR ("Non-communicable Chronic Diseases*"[Title/Abstract])) OR ("Noninfectious Diseases*"[Title/Abstract])) OR ("Noncommunicable Diseases"[Mesh])) OR ("Type 2 diabetes mellitus*"[Title/Abstract])) OR ("Type 2 diabetes*"[Title/Abstract])) OR ("Type 2 DM*"[Title/Abstract])) OR ("Diabetes*"[Title/Abstract])) OR ("Diabetes Mellitus, Type 2"[Mesh])) OR (Hypertension*[Title/Abstract])) OR (systemic hypertension*[Title/Abstract])) OR ("Hypertension"[Mesh])) OR (Asthma*[Title/Abstract])) OR ("Bronchial asthma*"[Title/Abstract])) OR ("Asthma"[Mesh])) OR (COPD*[Title/Abstract])) OR ("Chronic obstructive pulmonary disease*"[Title/Abstract])) OR ("Pulmonary Disease, Chronic Obstructive"[Mesh])) OR (CAD[Title/Abstract])) OR ("Coronary artery disease*"[Title/Abstract])) OR ("ischemic heart disease*"[Title/Abstract])) OR ("Stable angina*"[Title/Abstract])) OR ("Coronary Artery Disease"[Mesh])) OR ("Angina, Stable"[Mesh])))	5	1950/1/1-2023/11/10
#15	(("SEAMS"[Title/Abstract]) OR ("Self-Efficacy for Appropriate Medication Use Scale*"[Title/Abstract]))) AND ((((((((((((((((((((((((("Non-communicable diseases*"[Title/Abstract]) OR ("non-infectious diseases*"[Title/Abstract])) OR ("Non-communicable Chronic Diseases*"[Title/Abstract])) OR ("Noninfectious Diseases*"[Title/Abstract])) OR ("Noncommunicable Diseases"[Mesh])) OR ("Type 2 diabetes mellitus*"[Title/Abstract])) OR ("Type 2 diabetes*"[Title/Abstract])) OR ("Type 2 DM*"[Title/Abstract])) OR ("Diabetes*"[Title/Abstract])) OR ("Diabetes Mellitus, Type 2"[Mesh])) OR (Hypertension*[Title/Abstract])) OR (systemic hypertension*[Title/Abstract])) OR ("Hypertension"[Mesh])) OR (Asthma*[Title/Abstract])) OR ("Bronchial asthma*"[Title/Abstract])) OR ("Asthma"[Mesh])) OR (COPD*[Title/Abstract])) OR ("Chronic obstructive pulmonary disease*"[Title/Abstract])) OR ("Pulmonary Disease, Chronic Obstructive"[Mesh])) OR (CAD[Title/Abstract])) OR ("Coronary artery disease*"[Title/Abstract])) OR ("ischemic heart disease*"[Title/Abstract])) OR ("Stable angina*"[Title/Abstract])) OR ("Coronary Artery Disease"[Mesh])) OR ("Angina, Stable"[Mesh])))	7	1950/1/1-2023/11/10

Resource : EMBASE SEARCH

Date of search: 19/11/2023 Time: 10:02:46

Sl.no.	Search	No. of articles
1	*medication compliance	5444
2	medication compliance.mp.	20435
3	exp *patient compliance/ or medication non adherence.mp.	11535
4	drug non adherence.mp.	40
5	drug non-adherence.mp.	10
6	medication persistence.mp.	298
7	2 or 4 or 6 or 8 or 10 or 12	26074
8	exp *questionnaire/ or questionnaire.mp.	471478

9	health survey.mp. or exp *health survey/	52737
10	healthcare survey.mp. or exp *health care survey/	816
11	scale.mp. or exp *Medication Adherence Rating Scale/ or exp *"Basel Assessment of Adherence to Immunosuppressive Medications Scale"/ or exp *Morisky Medication Adherence Scale/	611729
12	(adherence to refill and medication scale).mp. [mp=title, abstract, heading word, drug trade name, original title, device manufacturer, drug manufacturer, device trade name, keyword heading word, floating subheading word, candidate term word]	1
13	brief medication questionnaire.mp.	87
14	compliance scale.mp.	99
15	adherence scale.mp.	2040
16	medication questionnaire.mp.	236
17	adherence report scale.mp.	229
18	15 or 17 or 19 or 21 or 23 or 25 or 27 or 29 or 31 or 33	993576

19	exp *cross validation/ or exp *validation study/ or exp *instrument validation/ or validation.mp. or exp *validation process/ or exp *validation therapy/	215311
20	validity.mp. or exp *validity/	84821
21	development.mp. or exp *development/	1379138
22	qualitative.mp. or exp *qualitative diagnosis/ or exp *qualitative analysis/ or exp *qualitative validity/ or exp *qualitative research/	143961
23	validation studies.mp. or exp *validation study/	8448
24	reproducibility of results.mp. or exp *reproducibility/	4504
25	36 or 38 or 40 or 42 or 44 or 46	1713587
26	non communicable disease.mp. or exp *non communicable disease/	6226
27	exp *chronic disease/ or non-infectious disease.mp. or exp *cardiovascular disease/	937473
28	diabetes mellitus.mp. or exp *diabetes mellitus/	535647

29	exp *white coat hypertension/ or exp *systolic hypertension/ or exp *diabetic hypertension/ or exp *hypertension/ or exp *hereditary hypertension/ or exp *essential hypertension/ or exp *borderline hypertension/ or hypertension.mp.	444146
30	exp *asthma/ or exp *allergic asthma/ or exp *severe asthma/ or asthma.mp.	127021
31	pulmonary disease.mp. or exp *lung disease/	369559
32	exp *chronic obstructive lung disease/ or chronic obstructive.mp.	75804
33	coronary artery disease.mp. or exp *coronary artery disease/	119264
34	angina.mp. or exp *angina pectoris/	38874
35	stable angina.mp. or exp *stable angina pectoris/	7235
36	49 or 51 or 53 or 55 or 57 or 59 or 61 or 63 or 65 or 67	1892075
37	13 and 34 and 47 and 68	418

Resource: Scopus

Date of search : 14/02/2024

Sl. No	Search	No. of Results
1	((((((((“medication adherence*”[title/abstract]) OR (“medication compliance*”[title/abstract])) OR (“medication non adherence*”[title/abstract])) medication noncompliance*”[title/abstract])) OR (“medication persistence*”[title/abstract])) OR (“medication adherence ”[mesh])) OR (“drug ence*”[title/abstract])) OR (“drug compliance*”[title/abstract])) OR (“drug noncompliance*”[title/abstract])) OR (“drug non adherence /abstract])	2,271
2	(((((((((((((((((survey*[title/abstract]) OR (“questionnaire development*”[title/abstract])) OR (scale*[title/abstract])) OR (“morisky scale*”[title/ abstract])) OR (“morisky*”[title/abstract])) OR (“mmas*[title/abstract])) OR (“hill bone*”[title/abstract])) OR (mars*[title/abstract])) OR (“medication adherence report scale*”[title/abstract])) OR (arms*[title/abstract])) OR (“brief medication questionnaire*”[title/abstract])) OR (“compliance scale*”[title/abstract])) OR (“adherence scale*”[title/abstract])) OR (“medication questionnaire*”[title/abstract])) OR (“self-efficacy for appropriate medication use scale*”[title/abstract])) OR (“medication use scale*”[title/abstract])) OR (“adherence report scale*”[title/abstract])) OR (“surveys and questionnaires”[mesh])) OR (“self report”[mesh])	1,99,675
3	((((((((((((((((((((((“non-communicable diseases*”[title/abstract]) OR (“non-infectious diseases*”[title/abstract])) OR (“non-communicable chronic diseases*”[title/abstract])) OR (“noninfectious diseases*”[title/abstract])) OR (“non communicable diseases”[mesh])) OR (“type 2 diabetes mellitus*”[title/abstract])) OR (“type 2 diabetes *”[title/abstract])) OR (“type 2 dm*”[title/abstract])) OR (“diabetes *”[title/abstract])) OR (“diabetes mellitus, type 2”[mesh])) OR (hypertension*[title/abstract])) OR (systemic AND hypertension*[title/ abstract])) OR (“hypertension”[mesh])) OR (asthma*[title/abstract])) OR (“bronchial asthma*”[title/abstract])) OR (“asthma”[mesh])) OR (copd*[title/abstract])) OR (“chronic obstructive pulmonary disease*”[title/abstract])) OR (“pulmonary disease, chronic obstructive”[mesh])) OR (cad[title/abstract])) OR (“coronary artery disease*”[title/ abstract])) OR (“ischemic heart disease*”[title/abstract])) OR (“stable angina*”[title/ abstract])) OR (“coronary artery disease”[mesh])) OR (“angina, stable”[mesh])	34,972
4	((((validation*[title/abstract]) OR (validity*[title/abstract])) OR (development*[title/ abstract])) OR (qualitative*[title/abstract])) OR (reproducibility of results*[mesh])	2,37,738
5	1 AND 2 AND 3 AND 4	778

Table B: Critical appraisal of articles

B1: JBI Critical appraisal for cross-sectional studies

Sl. No.	Author, Year	Q1	Q2	Q3	Q4	Q5	Q6	Q7	Q8	Total score (Out of 8)	Overall appraisal (Include / Exclude/ Seek further info)
1.	Fadhilah AN et al.,2019[1]	1	1	1	1	1	0	1	1	7	Include
2.	Al Khazaz H et.al., 2010[2]	1	1	0	1	0	0	1	1	5	Include
3.	Al Qerem W et al.,2022[3]	1	1	0	1	0	0	1	1	5	Include
4.	Alammari G et al.,2021[4]	1	1	0	1	0	0	1	1	5	Include
5.	Alhalaiqa F et al.,2014[5]	1	1	0	1	0	0	1	1	5	Include
6.	Alhazzani H et al.,2021[6]	1	1	0	1	0	0	1	1	5	Include
7.	Anbazhakan S et al.,2016[7]	1	1	1	1	0	0	1	1	6	Include
8.	Anuradha HV et al.,2022[8]	1	1	0	1	0	0	1	1	5	Include
9.	Arikan H et al., 2018[9]	1	1	0	1	0	0	1	1	5	Include
10.	Ashur S T et al., 2015[10]	1	1	0	1	0	0	1	1	5	Include
11.	Axelsson M et al.2016[11]	1	1	1	1	1	0	1	1	7	Include
12.	Arnet I et al.,2015[12]	1	1	1	1	0	0	1	1	6	Include
13.	Bailey S et al.,2015[13]	1	1	1	0	0	0	1	1	5	Include
14.	Ayoub D et al., 2019[14]	1	1	1	1	1	0	1	1	7	Include
15.	Dehghan M et al.,2015[15]	1	1	1	1	0	0	1	1	6	Include
16.	Ben A J et al.,2012[16]	1	1	1	1	1	0	1	1	7	Include
17.	Bharmal M et al.,2009[17]	1	1	1	0	0	0	1	1	5	Include
18.	Bou Serhal R et al.,2018[18]	1	1	1	1	1	0	1	1	7	Include
19.	Cabral A C et al.,2017[19]	1	1	1	1	0	0	1	1	6	Include
20.	Cabral A C et al.,2023[20]	1	1	1	0	0	0	1	1	5	Include
21.	Chan A H Y et al.,2020[21]	1	1	0	0	0	0	1	1	4	Include
22.	Chan A H Y et al.,2019[22]	1	1	1	0	0	0	1	1	5	Include

23.	Chatziefstratiou A et al.,2019[23]	1	1	1	1	0	0	1	1	6	Include
24.	Al abboud S A et al.,2016[24]	1	1	1	1	0	0	1	1	6	Include
25.	Hacihasanoglu R et al.,2012[25]	1	1	1	1	0	0	1	1	6	Include
26.	Chen P F et al.,2020[26]	1	1	1	1	0	0	1	1	6	Include
27.	Cheong AT et al.,2015[27]	1	1	1	1	0	0	1	1	6	Include
28.	Chung W W et al., 2014[28]	1	1	1	1	0	0	1	1	6	Include
29.	Fongwa M N et al.,2015[29]	1	1	0	1	0	0	1	1	5	Include
30.	Gallagher B D et al,2014[30]	1	1	0	1	0	0	1	1	5	Include
31.	Gokdogan F et al.,2017[31]	1	1	0	1	0	0	1	1	5	Include
32.	Asilar R H et al.,2013[32]	1	1	1	1	0	0	1	1	6	Include
33.	Hatah E et al., 2020[33]	1	1	1	1	0	0	1	1	6	Include
34.	Ibrahim L et al.,2020[34]	1	1	1	1	0	0	1	1	6	Include
35.	Iranpour A et al.,2022[35]	1	1	0	1	0	0	1	1	5	Include
36.	Islam M A et al.,2023[36]	1	1	1	0	0	0	1	1	5	Include
37.	Janezic A et al.,2017[37]	1	1	0	0	0	0	1	1	4	Include
38.	Jank S et al.,2009[38]	1	1	0	0	0	0	1	1	4	Include
39.	Jimenez K et al.,2016[39]	1	1	1	0	0	0	1	1	5	Include
40.	Karademir M et al.,2009[40]	1	1	1	0	0	0	1	1	5	Include
41.	Karbownik MS et al.,2019[41]	1	1	1	1	0	0	1	1	6	Include
42.	Kim M T et al.,1999[42]	1	1	1	1	0	0	1	1	6	Include
43.	Kim J H et al.,2014[43]	1	1	1	0	0	0	1	1	5	Include
44.	KIM C J et al.,2016[44]	1	1	1	1	0	0	1	1	6	Include
45.	Savoldelli V K et al.,2012[45]	1	1	1	0	1	1	1	1	7	Include
46.	Koschack J et al.,2010[46]	1	1	1	0	0	0	1	1	5	Include
47.	Kripalani S et al.,2009[47]	1	1	1	0	0	0	1	1	5	Include

48.	Kristina S A et al.,2019[48]	1	1	1	1	0	0	1	1	6	Include
49.	Krousel-Wood M et al.,2013[49]	1	1	1	0	1	1	1	1	7	Include
50.	Krousel-Wood M et al.,2019[50]	1	1	1	1	1	1	1	1	8	Include
51.	Lee S et al.,2017[51]	1	1	1	0	0	0	1	1	5	Include
52.	Lee S et al.,2019[52]	1	1	1	1	1	0	1	1	7	Include
53.	Liberato A C S et al., 2020[53]	1	1	1	1	0	0	1	1	6	Include
54.	Polanska B J et al., 2016[54]	1	1	1	0	0	0	1	1	5	Include
55.	Ladova K et al., 2014[55]	1	1	1	0	0	0	1	1	5	Include
56.	Laghousi D et al.,2021[56]	1	1	1	0	0	0	1	1	5	Include
57.	Lai P SM et al.,2020[57]	1	1	1	1	0	0	1	1	6	Include
58.	Mahmoud M A et al.,2021[58]	1	1	1	0	0	0	1	1	5	Include
59.	Maryem A et al.,2023[59]	1	1	1	0	0	0	1	1	5	Include
60.	Lee W Y et al.,2013[60]	1	1	1	1	0	0	1	1	6	Include
61.	Liberato A C S et al.,2016[61]	1	1	1	0	0	0	1	1	5	Include
62.	Mahler C et al.,2010[62]	1	1	1	0	0	0	1	1	5	Include
63.	Martinez-Perez P et al.,2021[63]	1	1	1	1	0	0	1	1	6	Include
64.	Mayberry L S et al.,2013[64]	1	1	1	0	0	0	1	1	5	Include
65.	Mehrabi S et al.,2023[65]	1	0	1	0	0	0	1	1	4	Include
66.	Moharamzad Y et al., 2015[66]	1	1	1	1	0	0	1	1	6	Include
67.	Mikhael E M et al.,2019[67]	1	1	1	1	0	0	1	1	6	Include
68.	Mora P A et al.,2011[68]	1	1	1	0	0	0	1	1	5	Include
69.	Morisky D E et al., 1986[69]	1	1	1	1	0	0	1	1	6	Include
70.	Mallah Z et al.,2019[70]	1	1	1	1	1	1	1	1	8	Include
71.	Muneswarao J et al.,2020[71]	1	1	1	0	0	0	1	1	5	Include
72.	Nakwafila O et al.,2022[72]	1	1	1	0	1	1	1	1	7	Include

73.	Nassar R I et al.2022[73]	1	1	1	0	0	1	1	1	6	Include
74.	Naqvi A A et al.,2018[74]	1	1	1	0	0	0	1	1	5	Include
75.	Naqvi A A et al.,2019[75]	1	1	1	0	0	0	1	1	5	Include
76.	Naqvi A et al.,2019[76]	1	1	1	0	0	0	1	1	5	Include
77.	Nguyen T H et al.,2021[77]	1	1	1	0	0	0	1	1	5	Include
78.	Okello S et al.,2016[78]	1	1	1	0	1	1	1	1	7	Include
79.	Pan J et al., 2020[79]	1	1	1	0	0	0	1	1	5	Include
80.	Cai Q et al.,2019[80]	1	1	1	0	0	0	1	1	5	Include
81.	Deoliveira-Filho A D et al.,2014[81]	1	1	1	1	0	0	1	1	6	Include
82.	Islam M A et al.,2023[82]	1	1	1	1	0	0	1	1	6	Include
83.	Naqvi A A et al.,2020[83]	1	1	1	0	1	1	1	1	7	Include
84.	Plaza V et al.,2016[84]	1	1	1	0	0	0	1	1	5	Include
85.	Risser J et al.,2007[85]	1	1	1	0	0	0	1	1	5	Include
86.	Ranasinghe P et al.,2018[86]	1	1	1	1	0	0	1	1	6	Include
87.	Saffari M et al., 2015[87]	1	1	1	0	0	0	1	1	5	Include
88.	Sakthong P et al., 2009[88]	1	1	1	1	0	0	1	1	6	Include
89.	Shakya R et al.,2022[89]	1	1	1	0	0	0	1	1	5	Include
90.	Shi Z et al.,2021[90]	1	1	1	1	0	0	1	1	6	Include
91.	Shima R et al., 2015[91]	1	1	1	0	0	0	1	1	5	Include
92.	Shrestha R et al.,2021[92]	1	1	1	0	0	0	1	1	5	Include
93.	Song Y et al., 2011[93]	1	1	1	1	0	0	1	1	6	Include
94.	Shin D S et al.,2013[94]	1	1	1	1	0	0	1	1	6	Include
95.	Svarstad B L et al.,1999[95]	1	1	1	0	0	0	1	1	5	Include
96.	Tan C S et al.,2018[96]	1	1	1	1	0	0	1	1	6	Include
97.	Tandon S et al.,2015[97]	1	1	1	1	0	0	1	1	6	Include

98.	Tangirala N C et al.,2020[98]	1	1	1	1	0	0	1	1	6	Include
99.	Toelle B G et al.,2020[99]	1	1	1	0	0	0	1	1	5	Include
100.	Tommelein E et al.,2014[100]	1	1	1	0	0	0	1	1	5	Include
101.	Uchmanowicz I et al.,2016[101]	1	0	1	0	0	0	1	1	4	Include
102.	Ueno H et al.,2018[102]	1	1	1	0	0	0	1	1	5	Include
103.	Unni E J et al.,2014[103]	1	1	1	0	0	0	1	1	5	Include
104.	Llorca C V et al.,2020[104]	1	1	0	0	1	1	1	1	6	Include
105.	Unni E J et al.,2009[105]	1	1	0	0	0	0	1	1	4	Include
106.	Van de Steeg N et al.,2009[106]	1	1	1	0	0	0	1	1	5	Include
107.	Wang Y et al.,2012[107]	1	1	1	1	0	0	1	1	6	Include
108.	Weinman J et al.,2019[108]	1	1	1	0	0	0	1	1	5	Include
109.	Wang Y H et al.,2023[109]	1	1	1	0	0	0	1	1	5	Include
110.	Wang Y et al.,2021[110]	1	1	1	0	0	0	1	1	5	Include
111.	Wells J et al., 2023[111]	1	1	1	0	0	0	1	1	5	Include
112.	Wells J S et al.,2022[112]	1	1	1	1	0	0	1	1	6	Include
113.	Wu J et al.,2020[113]	1	1	1	1	0	0	1	1	6	Include
114.	Zongo A et al.,2015[114]	1	1	1	0	1	1	1	1	7	Include
115.	Zongo A et al.,2016[115]	1	1	1	0	1	1	1	1	7	Include
116.	Chung W W et al.,2015[116]	1	1	1	1	0	0	1	1	6	Include
117.	Dehghan M et al.,2011[117]	1	1	1	1	0	0	1	1	6	Include
118.	Lomper K et al.,2018[118]	1	1	1	1	0	0	1	1	6	Include
119.	Ma C et al.,2011[119]	1	1	1	1	0	0	1	1	6	Include
120.	Mathias S D et al.,2001[120]	1	1	1	1	0	0	1	1	6	Include
121.	Mostafavi F et al.,2016[121]	1	1	1	1	0	0	1	1	6	Include
122.	Ogedegbe G et al.,2003[122]	1	1	1	1	0	0	1	1	6	Include

123.	Prado J C et al.,2007[123]	1	1	1	1	1	0	1	1	7	Include
124.	Tordera M P et al.,2009[124]	1	0	1	1	0	0	1	1	5	Include
125.	Ruiz M A et al.,2008[125]	1	1	1	0	0	0	1	1	5	Include
126.	Hahn S R et al.,2008[126]	1	1	1	0	1	0	1	1	6	Include
127.	Matza L S et al.,2008[127]	1	1	1	0	0	0	1	1	5	Include
128.	Matza L S et al.,2009[128]	1	1	1	0	0	0	1	1	5	Include
129.	Atkinson M J et al.,2004[129]	1	1	1	0	0	0	1	1	5	Include
130.	Atkinson M J et al.,2005[130]	1	1	1	0	0	0	1	1	5	Include
131.	Cohen J L et al.,2009[131]	1	1	1	1	0	0	1	1	6	Include
132.	George J et al.,2005[132]	1	1	1	0	0	0	1	1	5	Include
133.	Horne R et al.,1999[133]	1	1	1	0	0	0	1	1	5	Include
134.	Wallston K A et al.,2007[134]	1	1	1	1	1	0	1	1	7	Include
135.	Buszko K et al., 2016[135]	1	1	1	1	0	0	1	1	6	Include
136.	Munoz Cobos F et al.,2024[136]	1	1	1	1	0	0	1	1	6	Include
137.	Soares S M et al.,2024[137]	1	1	1	1	0	0	1	1	6	Include

B2: JBI Critical appraisal for Randomized control trials

Sl. No.	Author, Year	Q1	Q2	Q3	Q4	Q5	Q6	Q7	Q8	Q9	Q10	Q11	Q12	Q13	Overall score (Out of 13)	Overall appraisal (Include/Exclude/Seek info
1.	Fernandez S et al.,2008[138]	1	0	1	0	0	1	0	1	1	1	1	1	1	9	Include
2.	Chen Y J et al.,2020[139]	0	0	1	0	0	1	0	1	1	1	1	1	1	8	Include
3.	Wetzels G et al.,2006[140]	1	0	1	0	0	1	0	1	1	1	1	1	1	9	Include

Table C: Scoring developed for JBI critical appraisal checklist

JBI Critical appraisal checklist(140) does not provide scoring for inclusion of articles. So, review team developed a scoring system as per available information in article in consultation with experts. The scoring system is given below:

JBI Critical appraisal checklist for cross sectional studies(140)**:**

Sl. No.	Question	Scoring<br>Yes (1) No (0)
1.	Were the criteria for inclusion in the sample clearly defined?	
2.	Were the study subjects and the setting described in detail?	
3.	Was the exposure measured in a valid and reliable way?	
4.	Were objective, standard criteria used for measurement of the condition?	
5.	# Were confounding factors identified?	
6.	# Were strategies to deal with confounding factors stated?	
7.	Were the outcomes measured in a valid and reliable way?	
8.	Was appropriate statistical analysis used?	

- The total score ≥ 4 was considered as a good quality study and was included in our review

If the study states that the confounding factors are cleared or adjusted, question 5 and 6 can be scored 1.

JBI Critical appraisal checklist for Randomized control trials(140)**:**

Sl. No.	Question	Scoring<br>Yes (1) No (0)
1.	Was true randomization used for assignment of participants to treatment groups?	
2.	Was allocation to treatment groups concealed?	
3.	Were treatment groups similar at the baseline?	
4.	Were participants blind to treatment assignment?	
5.	Were treatment groups treated identically other than the intervention of interest?	
6.	Were those delivering the treatment blind to treatment assignment?	
7.	Were outcome assessors blind to treatment assignment?	
8.	Were outcomes measured in the same way for treatment groups?	

9.	Were outcomes measured in a reliable way	
10.	Was follow up complete and if not, were differences between groups in terms of their follow up adequately described and analyzed?	
11.	Were participants analyzed in the groups to which they were randomized?	
12.	Was appropriate statistical analysis used?	
13.	Was the trial design appropriate and any deviations from the standard RCT design (individual randomization, parallel groups) accounted for in the conduct and analysis of the trial?	

- The total score ≥ 6 was considered as a good quality study and was included in our review

Table D: List of excluded studies and reasons for exclusion

Sl. No.	Excluded articles	Reason for exclusion
**1.**	Alsous et al.,2017	Not meeting inclusion criteria
**2.**	Islam M A et al.,2021	Not meeting inclusion criteria
**3.**	Bagcivan et al.,2015	Aim/objective unrelated
**4.**	Cai Q et al.,2023	Focus not on development/ validation/ facilitators/ barriers of adherence
**5.**	Fond et al., 2016	Not meeting inclusion criteria
**6.**	Garfield et al., 2012	Not meeting inclusion criteria
**7.**	Fialko et al., 2008	Not meeting inclusion criteria
**8.**	Bekalu et al.,2016	Duplication
**9.**	Margolis et al., 2020	Not meeting inclusion criteria
**10.**	Muller S et al., 2015	Not meeting inclusion criteria
**11.**	Garcia Marcos et al., 2016	Not meeting inclusion criteria
**12.**	Nguyen.T et al., 2019	Not meeting inclusion criteria
**13.**	Brooks C M et al., 1994	Not providing adequate quality indicators
**14.**	Pedrosa et al., 2016	Not providing adequate quality indicators
**15.**	Qi Muge et al., 2022	Not meeting inclusion criteria
**16.**	Shiyanbola et al., 2018	Focus not on development/validation/facilitators/barriers of adherence
**17.**	Pandey et al., 2015	Not meeting inclusion criteria

**18.**	Moon et al.,2017	Not meeting inclusion criteria
**19.**	Lambert E V et al., 2006	Not providing adequate quality indicators
**20.**	Salgado T et al.,2013	Not meeting inclusion criteria
**21.**	Treibich et al., 2017	Aim/objective unrelated
**22.**	Vincze et al., 2020	Aim/objective unrelated
**23.**	Zschocke et al., 2014	Aim/objective unrelated

Table E: Psychometric properties of translational studies of scales

Self-report adherence Scale	No. of translational studies	Response rate (%)	Internal consistency	Sensitivity (%)	Specificity (%)	Feasibility	Stable over time (Pearson's/ Spearman's)	Correlation (Criterion validity)	Comparison measure of adherence	Sample size
MMAS 8	24	63.8 - 99	α = 0.41-0.83	32 – 92.8	22.3 – 88.5	NR	Spearman's rho: −0.431-0.94, Pearson's r = -0.16 – 0.92	Significant	MMAS 4, HbA1c, BP, MA-VAS, HBTS-English, ACT, SGRQ, Four-compartment Med Signals pillbox, HKT, MAT,POEMS, BMQ, Pill count	62 - 937
BMQ	12	NR	α = 0.6 -0.93	77 - 80	58 - 100	Missing value 0.03%, Feasible	Pearson's, r=0.28 -0.78	Significant	BMQ Arabic, MMAS 4, BMQ subscales, ARMS, HbA1c, MEMS, Pill count, BP, HBM	43 - 612
GMAS	10	85.5 - 91.6	α = 0.66 – 0.94	75.41–94.06	72.55 – 87.89	NR	Spearman's rho = 0.54 - 0.99, Pearson's, r = 0.537 - 0.861	Significant	MARS-5, ARMS, BMQ - Specific	119 - 500
MMAS 4/ MGT/ MAQ	8	81.4 – 91.8	α = 0.25 – 0.73	27.8 - 73.5	36 – 93.9	NR	Spearman's rho = 0.425 – 0.597, Pearson's, r=0.28	Significant	BMQ, MARS-5, HbA1c, Pill count	62 - 6237
MARS-5	5	52.7 – 86.6	α = 0.54 – 0.8	8.5 - 53	57 – 97.4	NR	Spearman's rho = 0.597, Pearson's, r = 0.61 - 0.83	Significant	MRA, SIMS-D, BMQ Arabic, MMS 4, MMAS 8, LDL-c	136 - 613
ARMS	5	72.73 – 89.1	α = 0.75 – 0.954	78.2	80.2	NR	Spearman's rho = -0.52 - −0.211	Significant	MMAS 8, HbA1c, SDSCA-MS,	100 - 314
HBCTS	5	87.6	α = 0.29 - 0.857	NR	NR	NR	Spearman's rho = 0.859	Significant	HBTS English version, BP	117 - 299
Hill Bone Compliance scale	4	92	α = 0.73 – 0.83	37%	63%	NR	NR	Significant	MMAS 8, BP, HBCS-English	68 - 400
MARS-10	3	84 - 87	α = 0.75 – 0.85	51 – 82.4	68 - 71	NR	NR	Significant	ACQ,CSS, Electronic adherence	294 - 452

TSQM	3	99	α = 0.67 – 0.84	NR	NR	NR	Spearman's rho = 0.34-0.46	NR	MMAS 8	190 -396
MALMAS	2	96.2	α = 0.565 - 0.654	88.9 - 92	29.6 - 32.8	NR	Spearman's rho = 0.715 – 0.797	Significant	MMAS 8,HbA1c	100 - 136
M-DRAW	2	NR	α = 0.74 – 0.873	NR	NR	NR	NR	NR	Nil	26 - 88
SEAMS	2	76.52	α = 0.768 - 0.886	NR	NR	NR	Pearson's r = 0.784	NR	Nil	204 - 264
SPUR - 27	2	24.7 - 82	α = 0.887-0.893	70.3	80.6	NR	NR	Significant	(MPR, CAT score, HbA1c, BMI, Anti glycemic agents	100 - 378
MAR-Scale	2	44 – 83.1	α > 0.7	NR	NR	NR	NR	NR	MMAS 4/ MGT, ACT	350 - 665
TAQPH	1	NR	α = 0.80	NR	NR	NR	Spearman's rho = 0.27 < 0.001	Significant	MMAS 8	330
MCQ	1	95.9	α = 0.782	NR	NR	NR	NR	Significant	HbA1c	232
LMAS	1	91.25	α = 0.854	71.1	94.74	NR	NR	NR	DMAS	182
PDSMS	1	NR	α =0.76	NR	NR	NR	NR	NR	Nil	62
MUSE	1	NR	α =0.93	NR	NR	NR	NR	NR	Nil	62
MASES-SF	1	NR	α =0.94	NR	NR	NR	NR	NR	MASES-R	150
MASES- R	1	NR	α = 0.95	NR	NR	NR	NR	NR	MEMS, MMAS 4	168
FATS	1	48	α = 0.78	NR	NR	NR	NR	NR	Hill-Bone Compliance Scale, 12-item CYHBPIQ,ESSI	147
A 14 scale	1	NR	α = 0.91	NR	NR	NR	NR	NR	MMAS 8	68
VAS	1	NR	NR	18 - 48	68 - 98	NR	NR	NR	Four-compartment Med Signals pillbox	149
DMAS 7	1	NR	α = 0.627	64.8	64.3	NR	Spearman's rho = 0.846	Significant	LMAS-14	300
TAI questionnaire	1	NR	α = 0.871	75.95–96.29	78.19 – 96.67	NR	Spearman's rho = 0.799	Significant	Asthma symptom control, PDC	120
MASES	1	NR	α > 0.92	NR	NR	NR	NR	NR	Nil	184
GAS	1	NR	α = 0.942	NR	NR	NR	Spearman-Brown coefficient = 0.939	Significant	MMAS 8	336
HBMA scale	1	NR	α = 0.80	NR	NR	NR	NR	NR	Hypertension Belief Scale, Hypertension Belief Scale and BP	525
IAQ	1	83	α = 0.73	73	80	NR	NR	NR	Inhaler adherence electronic monitor	74
SR-4scale	1	70.6	NR	NR	NR	NR	NR	Significant	HbA1c	156
HBM -based questionnaire	1	NR	α = 0.68	NR	NR	NR	NR	Significant	BMQ	126
ASK-20 scale	1	NR	α = 0.76	NR	NR	NR	NR	Significant	MMAS 4, patient reported generic health status measure (SF-12), and AQLQ, PDC	112
ASK -12 Scale	1	NR	α = 0.75	NR	NR	NR	NR	Significant	MMAS 4, 12-Item SF-12, Mini-AQLQ, ADS, and PDC	112
ASCD	1	97.09	α = 0.739	NR	NR	NR	NR	NR	Nil	413
ITBQ	1	NR	α = 0.613	NR	NR	NR	NR	NR	Nil	262
SMAQ	1	NR	α = 0.63	75.30	29.50	NR	Pearson's, r = 0.560	Significant	MMAS 4, BP	117

Abbreviations: MCQ- Medication Compliance Questionnaire**, MMAS-** Morisky Medication Adherence Scale**, MA-VAS-** Medication adherence Visual Analog Scale**, HBCTS-**Hill-Bone compliance to high blood pressure therapy scale**, ACT-** Asthma Control Test**, SGRQ-** Saint George Respiratory Questionnaire**, HKT-** Hypertension Knowledge Test**, MAT-**Measurement of Adherence to the Treatment**, POEMS-** Polypharmacy electronic monitoring system**, BMQ-**Beliefs about Medicines Questionnaire, **MARS**- Medication Adherence Report Scale, **MRA--** Medication Refill Adherence**, SIMS-D-**Satisfaction with Information about Medicines Scale**, LDL-c–** Low density lipoprotein**, BP**- Blood pressure**, HBM-** Health belief model based questionnaire**, ARMS-**Adherence to Refills and Medications Scale**, SDSCA-MS-** Summary of Diabetes Self-Care Activities medications subscale, **SEAMS**- Self-Efficacy for Appropriate Medication Use Scale, **TAQPH** - Treatment Adherence Questionnaire for Patient with Hypertension, **MGT**- Morisky-Green test, **MAQ-** Medication Adherence Questionnaire, **TSQM**- Treatment Satisfaction Questionnaire for Medication, **LMAS-** Lebanese Medication Adherence Scale, **PDSMS-** Perceived Diabetes Self-Management Scale, **MUSE-** Medication Understanding and Use Self Efficacy Scale, **DMAS-** Diabetes Medication Adherence Scale**, MASES-SF** -Medication Adherence Self-Efficacy Scale-Short Form , **HBCTS-** Hill-Bone compliance to high blood pressure therapy scale**, MALMAS-** Malaysian Medication Adherence Scale, **MASES- R-** Revised Medication Adherence Self-Efficacy Scale, **MEMS-**Medication Event Monitoring system**, FATS-** Facilitators of and Barriers to Adherence to Hypertension Treatment Scale, **CYHBPIQ-** Your High Blood Pressure IQ**, ESSI** -Enhancing Recovery From Coronary Heart Disease Social Support Inventory , **GMAS-** General Medication Adherence Scale, **VAS-** Visual Analogue Scale, **M-DRAW-** Modified Drug Adherence Work-up Tool, **ACQ-** Asthma Control Questionnaire, **CSS**- COPD Severity Score, **TAI-** Test of Adherence to Inhalers, **PDC-** Proportion of days covered, **GAS-** General Adherence Scale, **ACT** -Asthma Control Test, **MAR-Scale-** Medication Adherence Reasons Scale , **HBMA-** Hill-Bone Medication Adherence, **MPR** -Medication Possession Ratio, **CAT-**COPD Assessment Test**, COPD**- Chronic Obstructive Pulmonary Disease, BMI- Body mass index, **IAQ-** Inhaler Adherence Questionnaire**, SR-** self-report, **HBM-** Health belief model, **ASK-** Adherence Starts with Knowledge, **AQLQ-** Asthma Quality of Life Questionnaire, **PDC-** Proportion of days covered, **Mini-AQLQ-** Mini Asthma Quality of Life Questionnaire, **ADS-** Appraisal of Diabetes Scale, **ASCD**- Adherence Scale in Chronic Diseases, **ITBQ**- Inhaled Therapy Beliefs Questionnaire, **SMAQ**- Simplified Medication Adherence Questionnaire, **NR-**Not Reported

REFERENCES

1. Abdullah NF, Khuan L, Theng CA, Sowtali SN, Juni MH. Effect of patient characteristics on medication adherence among patients with type 2 diabetes mellitus: a cross-sectional survey. Contemp Nurse. 2019 Feb;55(1):27–37.

2. Al-Qazaz HK, Hassali MA, Shafie AA, Sulaiman SA, Sundram S, Morisky DE. The eight-item Morisky Medication Adherence Scale MMAS: translation and validation of the Malaysian version. Diabetes Res Clin Pract. 2010 Nov;90(2):216–21.

3. Al-Qerem W, Al Bawab AQ, Abusara O, Alkhatib N, Horne R. Validation of the Arabic version of medication adherence report scale questionnaire and beliefs about medication -specific questionnaire: A factor analysis study. PloS One. 2022;17(4):e0266606.

4. Alammari G, Alhazzani H, AlRajhi N, Sales I, Jamal A, Almigbal TH, et al. Validation of an Arabic Version of the Adherence to Refills and Medications Scale (ARMS). Healthc Basel Switz. 2021 Oct 24;9(11).

5. Alhalaiqa F, Masa'Deh R, Batiha AM, Deane K. Validity of Arabic Version of Beliefs About Medication Questionnaire. Clin Nurs Res. 2015 Oct;24(5):539–55.

6. Alhazzani H, AlAmmari G, AlRajhi N, Sales I, Jamal A, Almigbal TH, et al. Validation of an Arabic Version of the Self-Efficacy for Appropriate Medication Use Scale. Int J Environ Res Public Health. 2021 Nov 15;18(22).

7. Anbazhagan S, Fathima F, Agrawal T, Misquith D. Psychometric Properties of Morisky Medication Adherence Scale (MMAS) in Known Diabetic and Hypertensive Patients in a Rural Population of Kolar District, Karnataka. Indian J Public Health Res Dev. 2016 Jan 1;7:250.

8. Anuradha H.V., Prabhu P.S., Kalra P. Development and Validation of a Questionnaire for Assessing Medication Adherence in Type 2 Diabetes Mellitus in India. Biomed Pharmacol J. 2022;15(1):363–7.

9. Arıkan H, Duman D, Kargın F, Ergin G, Horne R, Karakurt S, et al. Cross-cultural Adaptation and Validation of Beliefs about Medicines Questionnaire on Asthma and Chronic Obstructive Pulmonary Disease Patients. Turk Thorac J. 2018 Jan;19(1):36–40.

10. Ashur ST, Shamsuddin K, Shah SA, Bosseri S, Morisky DE. Reliability and known-group validity of the Arabic version of the 8-item Morisky Medication Adherence Scale among type 2 diabetes mellitus patients. East Mediterr Health J Rev Sante Mediterr Orient Al-Majallah Al-Sihhiyah Li-Sharq Al-Mutawassit. 2015 Dec 13;21(10):722–8.

11. Axelsson M, Ekerljung L, Lundbäck B, Lötvall J. Personality and unachieved treatment goals related to poor adherence to asthma medication in a newly developed adherence questionnaire - a population-based study. Multidiscip Respir Med. 2016;11:42.

12. Arnet I, Metaxas C, Walter PN, Morisky DE, Hersberger KE. The 8-item Morisky Medication Adherence Scale translated in German and validated against objective and subjective polypharmacy adherence measures in cardiovascular patients. J Eval Clin Pract. 2015 Apr;21(2):271–7.

13. Bailey SC, Annis IE, Reuland DS, Locklear AD, Sleath BL, Wolf MS. Development and evaluation of the Measure of Drug Self-Management. Patient Prefer Adherence. 2015;9:1101–8.

14. Ayoub D, Mroueh L, El-Hajj M, Awada S, Rachidi S, Zein S, et al. Evaluation of antidiabetic medication adherence in the Lebanese population: development of the Lebanese Diabetes Medication Adherence Scale. Int J Pharm Pract. 2019 Oct;27(5):468–76.

15. Dehghan M, Dehghan-Nayeri N, Iranmanesh S. Translation and validation of the Persian version of the treatment adherence questionnaire for patients with hypertension. ARYA Atheroscler. 2016 Mar;12(2):76–86.

16. Ben AJ, Neumann CR, Mengue SS. The Brief Medication Questionnaire and Morisky-Green test to evaluate medication adherence. Rev Saude Publica. 2012 Apr;46(2):279–89.

17. Bharmal M, Payne K, Atkinson MJ, Desrosiers MP, Morisky DE, Gemmen E. Validation of an abbreviated Treatment Satisfaction Questionnaire for Medication (TSQM-9) among patients on antihypertensive medications. Health Qual Life Outcomes. 2009 Apr 27;7:36.

18. Bou Serhal R, Salameh P, Wakim N, Issa C, Kassem B, Abou Jaoude L, et al. A New Lebanese Medication Adherence Scale: Validation in Lebanese Hypertensive Adults. Int J Hypertens. 2018;2018:3934296.

19. Cabral AC, Moura-Ramos M, Castel-Branco M, Fernandez-Llimos F, Figueiredo IV. Cross-cultural adaptation and validation of a European Portuguese version of the 8-item Morisky medication adherence scale. Rev Port Cardiol. 2018 Apr;37(4):297–303.

20. Cabral AC, Lavrador M, Castel-Branco M, Figueiredo IV, Fernandez-Llimos F. Development and validation of a Medication Adherence Universal Questionnaire: the MAUQ. Int J Clin Pharm. 2023 Aug;45(4):999–1006.

21. Chan AHY, Vervloet M, Lycett H, Brabers A, van Dijk L, Horne R. Development and validation of a self-report measure of practical barriers to medication adherence: The medication practical barriers to adherence questionnaire (MPRAQ). Br J Clin Pharmacol. 2021 Nov;87(11):4197–211.

22. Chan AHY, Horne R, Hankins M, Chisari C. The Medication Adherence Report Scale: A measurement tool for eliciting patients' reports of nonadherence. Br J Clin Pharmacol. 2020 Jul;86(7):1281–8.

23. Chatziefstratiou A, Giakoumidakis K, Fotos NV, Baltopoulos G, Brokalaki H. Scales for assessing medication adherence in patients with hypertension. Br J Nurs Mark Allen Publ. 2019 Nov 28;28(21):1388–92.

24. Al Abboud SA, Ahmad S, Bidin MBL, Ismail NE. Validation of Malaysian Versions of Perceived Diabetes Self-Management Scale (PDSMS), Medication Understanding and Use Self-Efficacy Scale (MUSE) and 8-Morisky Medication Adherence Scale (MMAS-8) Using Partial Credit Rasch Model. J Clin Diagn Res JCDR. 2016 Nov;10(11):LC01–5.

25. Hacihasanoğlu R, Gözüm S, Capik C. Validity of the Turkish version of the medication adherence self-efficacy scale-short form in hypertensive patients. Anadolu Kardiyol Derg AKD Anatol J Cardiol. 2012 May;12(3):241–8.

26. Chen PF, Chang EH, Unni EJ, Hung M. Development of the Chinese Version of Medication Adherence Reasons Scale (ChMAR-Scale). Int J Environ Res Public Health. 2020 Aug;17(15):5578.

27. Cheong AT, Tong SF, Sazlina SG. Validity and reliability of the Malay version of the Hill-Bone compliance to high blood pressure therapy scale for use in primary healthcare settings in Malaysia: A cross-sectional study. Malays Fam Physician Off J Acad Fam Physicians Malays. 2015;10(2):36–44.

28. Siew Siang C, Lai P, Tan CH, Chan SP, Chung W, Morisky D. The development and validation of the malaysian medication Adherence scale (MALMAS) among patients with 2 type diabetes in Malaysia. Int J Pharm Pharm Sci. 2013 Jan 1;5:790–4.

29. Fongwa MN, Nandy K, Yang Q, Hays RD. The Facilitators of and Barriers to Adherence to Hypertension Treatment Scale. J Cardiovasc Nurs. 2015 Dec;30(6):484–90.

30. Gallagher BD, Muntner P, Moise N, Lin JJ, Kronish IM. Are two commonly used self-report questionnaires useful for identifying antihypertensive medication nonadherence? J Hypertens. 2015 May;33(5):1108–13.

31. Gökdoğan F, Kes D. Validity and reliability of the Turkish Adherence to Refills and Medications Scale. Int J Nurs Pract. 2017 Oct;23(5).

32. Hacıhasanoğlu Aşılar R, Gözüm S, Çapık C, Morisky DE. Reliability and validity of the Turkish form of the eight-item Morisky medication adherence scale in hypertensive patients. Anadolu Kardiyol Derg AKD Anatol J Cardiol. 2014 Dec;14(8):692–700.

33. Hatah E, Rahim N, Makmor-Bakry M, Mohamed Shah N, Mohamad N, Ahmad M, et al. Development and validation of Malaysia Medication Adherence Assessment Tool (MyMAAT) for diabetic patients. PloS One. 2020;15(11):e0241909.

34. Ibrahim L, Ibrahim L, Hallit S, Salameh P, Sacre H, Akel M, et al. Validation of the Lebanese Medication Adherence Scale among Lebanese diabetic patients. Int J Clin Pharm. 2021 Aug;43(4):918–27.

35. Iranpour A, Sarmadi V, Alian Mofrad A, Mousavinezhad SA, Mousavinezhad SM, Mohammad Alizadeh F, et al. The Persian version of the 8-item Morisky Medication Adherence Scale (MMAS-8): can we trust it? J Diabetes Metab Disord. 2022 Jun;21(1):835–40.

36. Islam MA, El-Dahiyat F, Nouri A, Alefan Q, Naqvi AA. Validation of the Arabic version of the general medication adherence scale in patients with type 2 diabetes mellitus in Jordan. Front Pharmacol. 2023;14:1194672.

37. Janežič A, Locatelli I, Kos M. Criterion validity of 8-item Morisky Medication Adherence Scale in patients with asthma. PloS One. 2017;12(11):e0187835.

38. Jank S, Bertsche T, Schellberg D, Herzog W, Haefeli WE. The A14-scale: development and evaluation of a questionnaire for assessment of adherence and individual barriers. Pharm World Sci PWS. 2009 Aug;31(4):426–31.

39. Jimenez K, Vargas C, Garcia K, Guzman H, Angulo M, Billimek J. Evaluating the Validity and Reliability of the Beliefs About Medicines Questionnaire in Low-Income, Spanish-Speaking Patients With Diabetes in the United States. Diabetes Educ. 2017 Feb;43(1):114–24.

40. Karademir M, Koseoglu IH, Vatansever K, Van Den Akker M. Validity and reliability of the Turkish version of the Hill-Bone compliance to high blood pressure therapy scale for use in primary health care settings. Eur J Gen Pract. 2009 Dec;15(4):207–11.

41. Karbownik MS, Jankowska-Polańska B, Horne R, Górski KM, Kowalczyk E, Szemraj J. Adaptation and validation of the Polish version of the Beliefs about Medicines Questionnaire among cardiovascular patients and medical students. PloS One. 2020;15(4):e0230131.

42. Kim MT, Hill MN, Bone LR, Levine DM. Development and Testing of the Hill-Bone Compliance to High Blood Pressure Therapy Scale. Prog Cardiovasc Nurs. 2000;15(3):90–6.

43. Kim JH, Lee WY, Hong YP, Ryu WS, Lee KJ, Lee WS, et al. Psychometric properties of a short self-reported measure of medication adherence among patients with hypertension treated in a busy clinical setting in Korea. J Epidemiol. 2014;24(2):132–40.

44. Kim CJ, Park E, Schlenk EA, Kim M, Kim DJ. Psychometric Evaluation of a Korean Version of the Adherence to Refills and Medications Scale (ARMS) in Adults With Type 2 Diabetes. Diabetes Educ. 2016 Apr;42(2):188–98.

45. Korb-Savoldelli V, Gillaizeau F, Pouchot J, Lenain E, Postel-Vinay N, Plouin PF, et al. Validation of a French version of the 8-item Morisky medication adherence scale in hypertensive adults. J Clin Hypertens Greenwich Conn. 2012 Jul;14(7):429–34.

46. Koschack J, Marx G, Schnakenberg J, Kochen MM, Himmel W. Comparison of two self-rating instruments for medication adherence assessment in hypertension revealed insufficient psychometric properties. J Clin Epidemiol. 2010 Mar;63(3):299–306.

47. Kripalani S, Risser J, Gatti ME, Jacobson TA. Development and evaluation of the Adherence to Refills and Medications Scale (ARMS) among low-literacy patients with chronic disease. Value Health J Int Soc Pharmacoeconomics Outcomes Res. 2009 Feb;12(1):118–23.

48. Kristina S.A., Putri L.R., Riani D.A., Ikawati Z., Endarti D. Validity of self-reported measure of medication adherence among diabetic patients in Indonesia. Int Res J Pharm. 2019;10(7):144–8.

49. Krousel-Wood M, Joyce C, Holt EW, Levitan EB, Dornelles A, Webber LS, et al. Development and evaluation of a self-report tool to predict low pharmacy refill adherence in elderly patients with uncontrolled hypertension. Pharmacotherapy. 2013 Aug;33(8):798–811.

50. Krousel-Wood M, Peacock E, Joyce C, Li S, Frohlich E, Re R, et al. A hybrid 4-item Krousel-Wood Medication Adherence Scale predicts cardiovascular events in older hypertensive adults. J Hypertens. 2019 Apr;37(4):851–9.

51. Lee S, Bae YH, Worley M, Law A. Validating the Modified Drug Adherence Work-Up (M-DRAW) Tool to Identify and Address Barriers to Medication Adherence. Pharm Basel Switz. 2017 Sep 8;5(3).

52. Lee S, Bae-Shaaw YH, Gogineni H, Worley MM, Law AV. Triple strength utility of the Modified Drug Adherence Work-Up (M-DRAW) tool in a veterans affairs outpatient diabetes clinic. Res Soc Adm Pharm RSAP. 2020 Jul;16(7):914–20.

53. Liberato ACS, São João TM, Jannuzzi FF, Landaas EJ, Wongchareon K, Rodrigues RCM. Treatment Satisfaction Questionnaire for Medication (TSQM version 1.4): Ceiling and Floor Effects, Reliability, and Known-Group Validity in Brazilian Outpatients With Hypertension. Value Health Reg Issues. 2020 Dec;23:150–6.

54. Jankowska-Polanska B, Uchmanowicz I, Chudiak A, Dudek K, Morisky DE, Szymanska-Chabowska A. Psychometric properties of the Polish version of the eight-item Morisky Medication Adherence Scale in hypertensive adults. Patient Prefer Adherence. 2016;10:1759–66.

55. Ladova K, Matoulkova P, Zadak Z, Macek K, Vyroubal P, Vlcek J, et al. Self-reported adherence by MARS-CZ reflects LDL cholesterol goal achievement among statin users: validation study in the Czech Republic. J Eval Clin Pract. 2014 Oct;20(5):671–7.

56. Laghousi D, Rezaie F, Alizadeh M, Asghari Jafarabadi M. The eight-item Morisky Medication Adherence Scale: validation of its Persian version in diabetic adults. Casp J Intern Med. 2021;12(1):77–83.

57. Lai PSM, Sellappans R, Chua SS. Reliability and Validity of the M-MALMAS Instrument to Assess Medication Adherence in Malay-Speaking Patients with Type 2 Diabetes. Pharm Med. 2020 Jun;34(3):201–7.

58. Mahmoud MA, Islam MA, Ahmed M, Bashir R, Ibrahim R, Al-Nemiri S, et al. Validation of the Arabic Version of General Medication Adherence Scale (GMAS) in Sudanese Patients with Diabetes Mellitus. Risk Manag Healthc Policy. 2021;14:4235–41.

59. Maryem A, Younes I, Yassmine M, Morad G, Karima B, Amal K, et al. Translation, cultural adaptation and validation of the General Medication Adherence Scale (GMAS) in moroccan patients with type-2 diabetes. BMC Nurs. 2023 Sep 4;22(1):302.

60. Lee WY, Ahn J, Kim JH, Hong YP, Hong SK, Kim YT, et al. Reliability and validity of a self-reported measure of medication adherence in patients with type 2 diabetes mellitus in Korea. J Int Med Res. 2013 Aug;41(4):1098–110.

61. Liberato ACS, Rodrigues RCM, São-João TM, Alexandre NMC, Gallani MCBJ. Satisfaction with medication in coronary disease treatment: psychometrics of the Treatment Satisfaction Questionnaire for Medication. Rev Lat Am Enfermagem. 2016 Jun 7;24:S0104-11692016000100334.

62. Mahler C, Hermann K, Horne R, Ludt S, Haefeli WE, Szecsenyi J, et al. Assessing reported adherence to pharmacological treatment recommendations. Translation and evaluation of the Medication Adherence Report Scale (MARS) in Germany. J Eval Clin Pract. 2010 Jun;16(3):574–9.

63. Martinez-Perez P, Orozco-Beltrán D, Pomares-Gomez F, Hernández-Rizo JL, Borras-Gallen A, Gil-Guillen VF, et al. Validation and psychometric properties of the 8-item Morisky Medication Adherence Scale (MMAS-8) in type 2 diabetes patients in Spain. Aten Primaria. 2021 Feb;53(2):101942.

64. Mayberry LS, Gonzalez JS, Wallston KA, Kripalani S, Osborn CY. The ARMS-D out performs the SDSCA, but both are reliable, valid, and predict glycemic control. Diabetes Res Clin Pract. 2013 Nov;102(2):96–104.

65. Mehrabi S., Shahryari F. Reliability and Validity of Persian Translation of Morisky Medication Adherence Scale (4-Item Version) in Asthmatic Patients. Shiraz E Med J. 2023;24(5):e135491.

66. Moharamzad Y, Saadat H, Nakhjavan Shahraki B, Rai A, Saadat Z, Aerab-Sheibani H, et al. Validation of the Persian Version of the 8-Item Morisky Medication Adherence Scale (MMAS-8) in Iranian Hypertensive Patients. Glob J Health Sci. 2015 Jan 1;7(4):173–83.

67. Mikhael EM, Hussain SA, Shawky N, Hassali MA. Validity and reliability of anti-diabetic medication adherence scale among patients with diabetes in Baghdad, Iraq: a pilot study. BMJ Open Diabetes Res Care. 2019;7(1):e000658.

68. Mora PA, Berkowitz A, Contrada RJ, Wisnivesky J, Horne R, Leventhal H, et al. Factor structure and longitudinal invariance of the Medical Adherence Report Scale-Asthma. Psychol Health. 2011 Jun;26(6):713–27.

69. Morisky DE, Green LW, Levine DM. Concurrent and predictive validity of a self-reported measure of medication adherence. Med Care. 1986 Jan;24(1):67–74.

70. Mallah Z, Hammoud Y, Awada S, Rachidi S, Zein S, Ballout H, et al. Validation of diabetes medication adherence scale in the Lebanese population. Diabetes Res Clin Pract. 2019 Oct;156:107837.

71. Muneswarao J, Hassali MA, Ibrahim B, Saini B, Naqvi AA, Hyder Ali IA, et al. Translation and validation of the Test of Adherence to Inhalers (TAI) questionnaire among adult patients with asthma in Malaysia. J Asthma Off J Assoc Care Asthma. 2021 Sep;58(9):1229–36.

72. Nakwafila O, Mashamba-Thompson T, Godi A, Sartorius B. A Cross-Sectional Study on Hypertension Medication Adherence in a High-Burden Region in Namibia: Exploring Hypertension Interventions and Validation of the Namibia Hill-Bone Compliance Scale. Int J Environ Res Public Health. 2022 Apr 6;19(7).

73. Nassar RI, Saini B, Obeidat NM, Basheti IA. Development and validation of the Adherence to Asthma Medication Questionnaire (AAMQ). Pharm Pract. 2022 Jun;20(2):2673.

74. Naqvi AA, Hassali MA, Rizvi M, Zehra A, Iffat W, Haseeb A, et al. Development and Validation of a Novel General Medication Adherence Scale (GMAS) for Chronic Illness Patients in Pakistan. Front Pharmacol. 2018;9:1124.

75. Naqvi AA, Hassali MA, Jahangir A, Nadir MN, Kachela B. Translation and validation of the English version of the general medication adherence scale (GMAS) in patients with chronic illnesses. J Drug Assess. 2019;8(1):36–42.

76. Naqvi AA, AlShayban DM, Ghori SA, Mahmoud MA, Haseeb A, Faidah HS, et al. Validation of the General Medication Adherence Scale in Saudi Patients With Chronic Diseases. Front Pharmacol. 2019;10:633.

77. Nguyen TH, Truong HV, Vi MT, Taxis K, Nguyen T, Nguyen KT. Vietnamese Version of the General Medication Adherence Scale (GMAS): Translation, Adaptation, and Validation. Healthc Basel Switz. 2021 Oct 29;9(11).

78. Okello S, Nasasira B, Muiru ANW, Muyingo A. Validity and Reliability of a Self-Reported Measure of Antihypertensive Medication Adherence in Uganda. PloS One. 2016;11(7):e0158499.

79. Pan J, Hu B, Wu L, Wang H, Lei T, Liu Z. The Translation, Reliability and Validity of the Chinese Version of the Hill-Bone Compliance to High Blood Pressure Therapy Scale in Adults with Hypertension. Patient Prefer Adherence. 2020;14:1853–60.

80. Cai Q, Ye L, Horne R, Bi J, Xu Q, Ye X, et al. Patients' adherence-related beliefs about inhaled steroids: application of the Chinese version of the Beliefs about Medicines Questionnaire-specific in patients with asthma. J Asthma Off J Assoc Care Asthma. 2020 Mar;57(3):319–26.

81. de Oliveira-Filho AD, Morisky DE, Neves SJF, Costa FA, de Lyra DPJ. The 8-item Morisky Medication Adherence Scale: validation of a Brazilian-Portuguese version in hypertensive adults. Res Soc Adm Pharm RSAP. 2014 Jun;10(3):554–61.

82. Islam MA, Iffat W, Imam S, Shakeel S, Rasheed A, Naqvi AA. Translation and validation of the Sindhi version of the general medication adherence scale in patients with chronic diseases. Front Pharmacol. 2023;14:1235032.

83. Naqvi AA, Mahmoud MA, AlShayban DM, Alharbi FA, Alolayan SO, Althagfan S, et al. Translation and validation of the Arabic version of the General Medication Adherence Scale (GMAS) in Saudi patients with chronic illnesses. Saudi Pharm J SPJ Off Publ Saudi Pharm Soc. 2020 Sep;28(9):1055–61.

84. Plaza V, Fernández-Rodríguez C, Melero C, Cosío BG, Entrenas LM, de Llano LP, et al. Validation of the "Test of the Adherence to Inhalers" (TAI) for Asthma and COPD Patients. J Aerosol Med Pulm Drug Deliv. 2016 Apr;29(2):142–52.

85. Risser J, Jacobson TA, Kripalani S. Development and psychometric evaluation of the Self-efficacy for Appropriate Medication Use Scale (SEAMS) in low-literacy patients with chronic disease. J Nurs Meas. 2007;15(3):203–19.

86. Ranasinghe P, Jayawardena R, Katulanda P, Constantine GR, Ramanayake V, Galappatthy P. Translation and Validation of the Sinhalese Version of the Brief Medication Questionnaire in Patients with Diabetes Mellitus. J Diabetes Res. 2018;2018:7519462.

87. Saffari M, Zeidi IM, Fridlund B, Chen H, Pakpour AH. A Persian Adaptation of Medication Adherence Self-Efficacy Scale (MASES) in Hypertensive Patients: Psychometric Properties and Factor Structure. High Blood Press Cardiovasc Prev Off J Ital Soc Hypertens. 2015 Sep;22(3):247–55.

88. Sakthong P, Chabunthom R, Charoenvisuthiwongs R. Psychometric properties of the Thai version of the 8-item Morisky Medication Adherence Scale in patients with type 2 diabetes. Ann Pharmacother. 2009 May;43(5):950–7.

89. Shakya R, Shrestha R, Shrestha S, Sapkota P, Gautam R, Rai L, et al. Translation, Cultural Adaptation and Validation of the Hill Bone Compliance to High Blood Pressure Therapy Scale to Nepalese Language. Patient Prefer Adherence. 2022;16:957–70.

90. Shi Z, Chang J, Ma X, Yin F, Ma M, Li W, et al. The Psychometric Properties of General Adherence Scale in Chinese (GAS-C) in Patients with Type 2 Diabetes Using Insulin. Diabetes Metab Syndr Obes Targets Ther. 2021;14:801–11.

91. Shima R, Farizah H, Majid HA. The 11-item Medication Adherence Reasons Scale: reliability and factorial validity among patients with hypertension in Malaysian primary healthcare settings. Singapore Med J. 2015 Aug;56(8):460–7.

92. Shrestha R, Sapkota B, Khatiwada AP, Shrestha S, Khanal S, Kc B, et al. Translation, Cultural Adaptation and Validation of General Medication Adherence Scale (GMAS) into the Nepalese Language. Patient Prefer Adherence. 2021;15:1873–85.

93. Song Y, Han HR, Song HJ, Nam S, Nguyen T, Kim MT. Psychometric evaluation of hill-bone medication adherence subscale. Asian Nurs Res. 2011 Sep;5(3):183–8.

94. Shin DS, Kim CJ. Psychometric evaluation of a Korean version of the 8-item Medication Adherence Scale in rural older adults with hypertension. Aust J Rural Health. 2013 Dec;21(6):336–42.

95. Svarstad BL, Chewning BA, Sleath BL, Claesson C. The brief medication questionnaire: A tool for screening patient adherence and barriers to adherence. Patient Educ Couns. 1999 Jun 1;37(2):113–24.

96. Tan CS, Hassali MA, Neoh CF, Saleem F, Horne R. Cultural Adaptation and Linguistic Validation of the Beliefs about Medicines Questionnaire in Malaysia. Value Health Reg Issues. 2018 May;15:161–8.

97. Tandon S, Chew M, Eklu-Gadegbeku CK, Shermock KM, Morisky DE. Validation and psychometric properties of the 8-item Morisky Medication Adherence Scale (MMAS-8) in Type 2 diabetes patients in sub-Saharan Africa. Diabetes Res Clin Pract. 2015 Nov;110(2):129–36.

98. Tangirala NC, O'Conor R, Wolf MS, Wisnivesky JP, Federman AD. Validity of the Medication Adherence Rating Scale for Adherence to Inhaled Corticosteroids among Older Adults with Asthma or Chronic Obstructive Pulmonary Disease. COPD. 2020 Feb;17(1):74–80.

99. Toelle BG, Marks GB, Dunn SM. Validation of the inhaler adherence questionnaire. BMC Psychol. 2020 Sep 3;8(1):95.

100. Tommelein E, Mehuys E, Van Tongelen I, Brusselle G, Boussery K. Accuracy of the Medication Adherence Report Scale (MARS-5) as a quantitative measure of adherence to inhalation medication in patients with COPD. Ann Pharmacother. 2014 May;48(5):589–95.

101. Uchmanowicz I, Jankowska-Polańska B, Chudiak A, Szymańska-Chabowska A, Mazur G. Psychometric evaluation of the Polish adaptation of the Hill-Bone Compliance to High Blood Pressure Therapy Scale. BMC Cardiovasc Disord. 2016 May 10;16:87.

102. Ueno H, Yamazaki Y, Yonekura Y, Park MJ, Ishikawa H, Kiuchi T. Reliability and validity of a 12-item medication adherence scale for patients with chronic disease in Japan. BMC Health Serv Res. 2018 Jul 31;18(1):592.

103. Unni EJ, Olson JL, Farris KB. Revision and validation of Medication Adherence Reasons Scale (MAR-Scale). Curr Med Res Opin. 2014 Feb;30(2):211–21.

104. Valdés Y Llorca C, Cortés-Castell E, Ribera-Casado JM, de Lucas-Ramos P, de Palacio-Guerrero LM, Mugarza-Borqué F, et al. Validation of self-reported adherence in chronic patients visiting pharmacies and factors associated with the overestimation and underestimation of good adherence. Eur J Clin Pharmacol. 2020 Nov;76(11):1607–14.

105. Unni EJ, Farris KB. Development of a new scale to measure self-reported medication nonadherence. Res Soc Adm Pharm RSAP. 2015 Jun;11(3):e133-143.

106. van de Steeg N, Sielk M, Pentzek M, Bakx C, Altiner A. Drug-adherence questionnaires not valid for patients taking blood-pressure-lowering drugs in a primary health care setting. J Eval Clin Pract. 2009 Jun;15(3):468–72.

107. Wang Y, Lee J, Toh MPHS, Tang WE, Ko Y. Validity and reliability of a self-reported measure of medication adherence in patients with Type 2 diabetes mellitus in Singapore. Diabet Med J Br Diabet Assoc. 2012 Sep;29(9):e338-344.

108. Weinman J, Ali I, Hodgkinson A, Canfield M, Jackson C. Pilot Testing Of A Brief Pre-Consultation Screener For Improving The Identification And Discussion Of Medication Adherence In Routine Consultations. Patient Prefer Adherence. 2019;13:1895–8.

109. Wang YH, Yang TM, Hung MS, Lin YC, Fang TP, Kuo TT, et al. The Intention of Inhaled Medication Adherence Scale (IMAS): The Development of a New Instrument for Assessing Inhaled Medication Adherence Among Patients with Chronic Obstructive Pulmonary Disease (COPD) Using Theory of Planned Behavior. Int J Chron Obstruct Pulmon Dis. 2023;18:1655–64.

110. Wang Y, Wang X, Wang X, Naqvi AA, Zhang Q, Zang X. Translation and validation of the Chinese version of the general medication adherence scale (GMAS) in patients with chronic illness. Curr Med Res Opin. 2021 May;37(5):829–37.

111. Wells J, Mahendran S, Dolgin K, Kayyali R. SPUR-27 - Psychometric Properties of a Patient-Reported Outcome Measure of Medication Adherence in Chronic Obstructive Pulmonary Disease. Patient Prefer Adherence. 2023;17:457–72.

112. Wells JS, El Husseini A, Okoh S, Jaffar A, Neely C, Crilly P, et al. SPUR: psychometric properties of a patient-reported outcome measure of medication adherence in type 2 diabetes. BMJ Open. 2022 Sep 6;12(9):e058467.

113. Wu J, Tao Z, Song Z, Zhang Y, Sun H, Wang J, et al. Validation and psychometric properties of the self-efficacy for Appropriate Medication Use Scale in elderly Chinese patients. Int J Clin Pharm. 2021 Jun;43(3):586–94.

114. Zongo A, Guénette L, Moisan J, Grégoire JP. Predictive Validity of Self-Reported Measures of Adherence to Noninsulin Antidiabetes Medication against Control of Glycated Hemoglobin Levels. Can J Diabetes. 2016 Feb;40(1):58–65.

115. Zongo A, Guénette L, Moisan J, Guillaumie L, Lauzier S, Grégoire JP. Revisiting the internal consistency and factorial validity of the 8-item Morisky Medication Adherence Scale. SAGE Open Med. 2016;4:2050312116674850.

116. Chung WW, Chua SS, Lai PSM, Morisky DE. The Malaysian Medication Adherence Scale (MALMAS): Concurrent Validity Using a Clinical Measure among People with Type 2 Diabetes in Malaysia. PloS One. 2015;10(4):e0124275.

117. dehghan nayeri N, Iranmanesh S. Validating the Persian Version of the Hill-Bone's Scale of "Compliance to High Blood Pressure Therapy." Br J Med Med Res. 2011 Jan 20;5:235–46.

118. Lomper K, Chabowski M, Chudiak A, Białoszewski A, Dudek K, Jankowska-Polańska B. Psychometric evaluation of the Polish version of the Adherence to Refills and Medications Scale (ARMS) in adults with hypertension. Patient Prefer Adherence. 2018;12:2661–70.

119. Ma C, Chen S, You L, Luo Z, Xing C. Development and psychometric evaluation of the Treatment Adherence Questionnaire for Patients with Hypertension. J Adv Nurs. 2012;68(6):1402–13.

120. Mathias SD, Warren EH, Colwell HH, Sung JC. A new treatment satisfaction measure for asthmatics: a validation study. Qual Life Res Int J Qual Life Asp Treat Care Rehabil. 2000;9(7):873–82.

121. Mostafavi F, Najimi A, Sharifirad G, Golshiri P. BELIEFS ABOUT MEDICINES IN PATIENTS WITH HYPERTENSION: THE INSTRUMENT VALIDITY AND RELIABILITY IN IRAN. Mater Socio-Medica. 2016 Jul 24;28(4):298–302.

122. Ogedegbe G, Mancuso CA, Allegrante JP, Charlson ME. Development and evaluation of a medication adherence self-efficacy scale in hypertensive African-American patients. J Clin Epidemiol. 2003 Jun;56(6):520–9.

123. Prado JC, Kupek E, Mion D. Validity of four indirect methods to measure adherence in primary care hypertensives. J Hum Hypertens. 2007 Jul;21(7):579–84.

124. Perpiñá Tordera M, Moragón EM, Fuster AB, Bayo AL, Císcar CP. Spanish Asthma Patients' Beliefs About Health and Medicines: Validation of 2 Questionnaires. Arch Bronconeumol Engl Ed. 2009 May 1;45(5):218–23.

125. Ruiz MA, Pardo A, Rejas J, Soto J, Villasante F, Aranguren JL. Development and validation of the "Treatment Satisfaction with Medicines Questionnaire" (SATMED-Q). Value Health J Int Soc Pharmacoeconomics Outcomes Res. 2008;11(5):913–26.

126. Hahn SR, Park J, Skinner EP, Yu-Isenberg KS, Weaver MB, Crawford B, et al. Development of the ASK-20 adherence barrier survey. Curr Med Res Opin. 2008 Jul;24(7):2127–38.

127. Matza LS, Yu-Isenberg KS, Coyne KS, Park J, Wakefield J, Skinner EP, et al. Further testing of the reliability and validity of the ASK-20 adherence barrier questionnaire in a medical center outpatient population. Curr Med Res Opin. 2008 Nov;24(11):3197–206.

128. Matza LS, Park J, Coyne KS, Skinner EP, Malley KG, Wolever RQ. Derivation and validation of the ASK-12 adherence barrier survey. Ann Pharmacother. 2009 Oct;43(10):1621–30.

129. Atkinson MJ, Sinha A, Hass SL, Colman SS, Kumar RN, Brod M, et al. Validation of a general measure of treatment satisfaction, the Treatment Satisfaction Questionnaire for Medication (TSQM), using a national panel study of chronic disease. Health Qual Life Outcomes. 2004 Feb 26;2:12.

130. Atkinson MJ, Kumar R, Cappelleri JC, Hass SL. Hierarchical construct validity of the treatment satisfaction questionnaire for medication (TSQM version II) among outpatient pharmacy consumers. Value Health J Int Soc Pharmacoeconomics Outcomes Res. 2005;8 Suppl 1:S9–24.

131. Cohen JL, Mann DM, Wisnivesky JP, Home R, Leventhal H, Musumeci-Szabó TJ, et al. Assessing the validity of self-reported medication adherence among inner-city asthmatic adults: the Medication Adherence Report Scale for Asthma. Ann Allergy Asthma Immunol Off Publ Am Coll Allergy Asthma Immunol. 2009 Oct;103(4):325–31.

132. George J, Mackinnon A, Kong DCM, Stewart K. Development and validation of the Beliefs and Behaviour Questionnaire (BBQ). Patient Educ Couns. 2006 Dec;64(1–3):50–60.

133. Horne R, Weinman J, Hankins M. The beliefs about medicines questionnaire: The development and evaluation of a new method for assessing the cognitive representation of medication. Psychol Health. 1999 Jan 1;14(1):1–24.

134. Wallston KA, Rothman RL, Cherrington A. Psychometric properties of the Perceived Diabetes Self-Management Scale (PDSMS). J Behav Med. 2007 Oct;30(5):395–401.

135. Buszko K, Obońska K, Michalski P, Kosobucka A, Jurek A, Wawrzyniak M, et al. The Adherence Scale in Chronic Diseases (ASCD). The power of knowledge: the key to successful patient — health care provider cooperation. Med Res J. 2016;1(1):37–42.

136. Muñoz-Cobos F, Aguiar-Leiva VP, Argüello-Suárez C, Colacicchi P, Calleja-Cartón LA, Leiva-Fernández F. Validation of an Inhaled Therapy Beliefs Questionnaire in Patients with Chronic Obstructive Pulmonary Disease. J Clin Med. 2024 Apr 15;13(8):2281.

137. Soares SM, Diniz MQ de A, Davino DMBMC, Albieri FB, Santos AS, Jesus EMS, et al. The Simplified Medication Adherence Questionnaire: validation of a Brazilian-Portuguese version in hypertensive adults. Front Pharmacol. 2024 Apr 11;15:1348917.

138. Fernandez S, Chaplin W, Schoenthaler AM, Ogedegbe G. Revision and validation of the medication adherence self-efficacy scale (MASES) in hypertensive African Americans. J Behav Med. 2008 Dec;31(6):453–62.

139. Chen YJ, Chang J, Yang SY. Psychometric Evaluation of Chinese Version of Adherence to Refills and Medications Scale (ARMS) and Blood-Pressure Control Among Elderly with Hypertension. Patient Prefer Adherence. 2020;14:213–20.

140. Wetzels G, Nelemans P, van Wijk B, Broers N, Schouten J, Prins M. Determinants of poor adherence in hypertensive patients: development and validation of the "Maastricht Utrecht Adherence in Hypertension (MUAH)-questionnaire." Patient Educ Couns. 2006 Dec;64(1–3):151–8.
