# Supplementary material for: Medication adherence scales in non-communicable diseases: A scoping review of design gaps, constructs and validation processes
Source: PLoS One. 2025 May 14;20(5):e0321423. doi: 10.1371/journal.pone.0321423 (PMC12077792; doi:10.1371/journal.pone.0321423)
Supplement: S4 Text — (PDF) [file pone.0321423.s004.pdf]

MEDICATION ADHERENCE SCALES USED IN NON-COMMUNICABLE DISEASES - A SCOPING REVIEW PROTOCOL

ABSTRACT

Introduction: The key contributor to the increasing burden of morbidity and mortality due to Non-Communicable-Diseases (NCDs) is poor control status of the patients, which is in turn due to poor medication adherence (MA). A reliable MA assessment method which is objective, valid, affordable, accessible and respects the linguistic and cultural diversity of the Indian population is crucial. There is an immediate requirement to methodically compile and analyze the data derived from the diverse medication adherence scales currently in use.

Objective: This scoping review is aimed at synthesizing the evidence concerning various medication adherence scales employed for select NCDs and to identify the gaps/ deficiencies in the scales.

Methods and Analysis: The scoping review will be guided by the Joanna Briggs Institute (JBI) methodological framework. Electronic databases, reference list of included articles, and scoping or systematic reviews identified from the search and grey literature will be searched. Studies published in the English language conducted since January 1950 to August 2023 will be included. Two reviewers will independently screen all articles, any conflicts between the reviewers, will be settled by a third reviewer. Critical appraisal of the screened-in articles would be done using JBI critical appraisal scales. The data will be compiled into a table and narrative summary that is consistent with the review's goal.

Keywords: medication adherence; non-communicable diseases; psychometric properties; self-report; scoping review

INTRODUCTION

Background

'Medication adherence' is defined as the extent to which a person's medication taking behavior corresponds with agreed recommendations from a health care provider.(1) Adherence to medication is a crucial part of patient care and indispensable for reaching clinical goals. The World Health Organization's (WHO) report on medication adherence, states that "increasing the effectiveness of adherence interventions may have a far greater impact on the health of the population than any improvement in specific medical treatment.(1) In developed countries, only 50% of the patients treated for chronic diseases adhere to the prescribed treatment and the problem in developing countries is greater.(2) The key contributor to the increasing burden of morbidity and mortality due to NCDs is the poor control status of the patients, which is in turn due to poor medication adherence.(3)

In India, non-adherence to medication varies from 18.7% to 74%, (3–6) which is assessed using scales validated in the Western population. NCDs contribute to around 38 million (68%) of all deaths globally and to about 5.87 million (60%) of all deaths in India (Burden of NCDs and their risk factors in India.(2) More than 30% of medicine-related hospital admissions occur due to medication non-adherence.(7,8) The persistent issue of medication non-adherence is a substantial obstacle in attaining improved health results for chronic illnesses. This problem leads to a significant increase in symptom severity, a more rapid deterioration in health-related quality of life, a rise in hospital admissions, mortality rates, and healthcare expenses.

The problem of non-adherence may arise due to patient-related factors (having negative beliefs or perceptions on medications, inadequate knowledge about therapy, changing routines due to festivals, fasting, frequent traveling or illness), condition-related factors (poor understanding of the disease, lack of visible symptoms), socioeconomic - related factors, therapy-related factors (complexity of treatment regime, pill burden, fear of side effects or treatment fatigue) and health system/ health care team related factors.(9) Beyond these, other barriers include a lack of belief in the need for treatment, stigma, and preference for alternative medicines. A study conducted in Bangalore by Thomas D et al.(10) reported, 39.14% not adherent due to specific beliefs about their treatment, 78.62% reported difficulty paying for the medication and 54.93% indicated that it is difficult to get a refill.

Evaluating medication adherence is crucial for determining the impact of treatment and the patient's response to therapy. This process also aids in identifying patients who are not adhering to their medication regimen and may need additional education and support to enhance their adherence. Understanding why patients discontinue their medications is vital in addressing those issues, thereby optimizing the effectiveness of the medication and enhancing the health outcomes of the patients.(11)

Furthermore, it's essential to detect medication non-adherence to prevent potential treatment effects from being underestimated. For healthcare professionals, accurately gauging medication adherence poses a challenge due to its association with fluctuating and evolving patient behaviors.(12) A robust assessment method is crucial. It needs to be not just reliable, but also objective, valid, economical, and readily accessible. However, a delicate equilibrium must be maintained between validity and cost-effectiveness. High-validity methods typically demand more resources, making

them more costly. This situation creates a trade-off where striving for validity may inadvertently increase expenses.(12)

Objective measures of adherence include pill counts, electronic monitoring, secondary database analysis, and biochemical measures which are often impractical and resource intensive. Subjective measures like self-reports and health care professional assessments rely on data which could be influenced by social desirability bias, recall bias and might not capture the complex cultural, and socioeconomic factors. Nevertheless, it has the benefits of being cost-effective, non-intrusive, user-friendly, easy to administer, and able to capture patient's concerns and thereby individualize adherence interventions.

Rationale

There is an urgent need to methodically compile and analyze the evidence derived from the different medication adherence scales currently in use for NCD adherence measurement. This would be done by taking into account their psychometric characteristics, including the delineation of quality benchmarks such as sensitivity, specificity, convergent validity, and reliability metrics. identify and address the deficiencies in the existing Medication Adherence (MA) scales. This scoping review, which is part of a research project aimed at developing and validating a medication adherence scale contextualized to the Indian setting, aims to synthesize the evidence concerning various medication adherence scales and to identify and address the deficiencies existing in them.

Review questions:

1. What is the existing evidence on the various medication adherence scales used in select NCDs [Type 2 Diabetes Mellitus (T2DM), Hypertension (HTN), Coronary Artery Disease (CAD), Bronchial asthma (BA)/Chronic Obstructive Pulmonary Disease (COPD)]?
2. What are the gaps/ deficiencies in the scales that are currently in use?

METHODS

The proposed scoping review will be conducted following the JBI methodology.(13,14) The reporting of the scoping review will follow the Preferred Reporting Items for Systematic Reviews and Meta-Analyses extension for Scoping Reviews (PRISMA-ScR).(15)

Eligibility criteria/ Inclusion criteria

Participants

Published studies of adults diagnosed with one or more of the following NCDs (T2DM, HTN, COPD/ BA, CAD), written in the English language on the development and/or validation of the adherence scale.

Concept

The review will consider the assessment of medication adherence using any medication adherence scales in select NCDs (T2DM, HTN, COPD/ Bronchial asthma, CAD). Medication adherence is defined as the extent to which a person's medication taking behavior corresponds with agreed recommendations from a health care provider.(1)

Context

This scoping review will consider medication adherence scales used and evaluated in the context of select NCDs (T2DM, HTN, COPD/ BA, CAD).

Types of evidence sources

This scoping review will consider quantitative studies, mixed methods studies, and systematic or scoping reviews. Quantitative designs include any experimental study designs (e.g., randomized controlled trials, non-randomized controlled trials, or other quasi-experimental studies, including before and after studies), and observational designs (e.g., descriptive studies, cohort studies, and cross-sectional studies). Mixed methods include quantitative and qualitative designs used for the validation of medication adherence scales.

Search strategy

The search strategy will aim to locate both published and unpublished primary studies and reviews. An initial search of MEDLINE (PubMed), Embase and Embase Classic, Cochrane CENTRAL Register of Clinical Trials, and APA PsycINFO will be conducted. The preliminary search strategy for MEDLINE (PubMed) and Embase is provided (See Appendix I). The Cochrane CENTRAL Register of Clinical Trials, APA PsycINFO, and Web of Science database will be searched further. The search for unpublished studies will include GreyNet, OpenGrey, and Shodhganga. A reference list of included articles and scoping or systematic reviews identified from the search will be searched. The following concepts were utilized to form the search strategy:

- Medication adherence
- Questionnaire
- Validation

- Non communicable diseases

The final search strategy structure using Boolean operators AND, OR and NOT will be used to search each database. Studies published in the English language conducted from January 1950 to August 2023 will be assessed for possible inclusion.

Study selection /Screening

After the search process, all found citations will be collated and imported into Zotero 6.0.30 (Corporation for Digital Scholarship and Roy Rosenzweig Center for History and New Media, VA, USA) referencing software, with any duplicates being eliminated. Two independent reviewers will scrutinize the titles and abstracts against the inclusion criteria for the scoping review and full texts of screened-in studies will be obtained. If the percentage of agreement between the two reviewers is less than 75%, consultation by a third reviewer would be done. Critical appraisal of the screened-in articles would be done using JBI critical appraisal scales.

Data Charting

Data from the papers selected for the review will be compiled following the scoping review methodology established by JBI methodology for scoping reviews. The 'descriptive-analytical' method within the narrative tradition will be utilized in data charting. The data extracted will include specific details about author(s), year and type of publication, setting, aims, study design, population demographics (eg, age, race, gender), and key findings relevant to the scoping review questions and objectives. The data extraction scale has been adapted from the guidelines provided by the JBI methodology for the scoping reviews format.(13) Two reviewers will independently map the studies using the data charting form into Microsoft Excel. They will then

convene to ensure their data extraction methods align with the research question and aim. A draft data extraction table is provided in Appendix II. The draft data extraction scale will be modified and revised as necessary during the process of extracting data. Missing or incomplete or unclear information would be coded as Not reported or Unclear and the corresponding authors will be contacted for further information or clarification of the data whenever required.

Synthesis and Presentation of Results

The data we extract will be displayed in a table format including author and year of publication, country, country income level, single/multicentric, objectives, type of study, research design, MA assessment scale, medication, disease evaluated, sample size etc. To represent the information flow throughout the review process, we will utilize a PRISMA flow diagram. We will compile and present the pertinent data as a descriptive summary, which will complement the results presented in tables and/or charts. A summation or appraisal of each paper will encompass the following elements: the author(s), the year it was published, the country where the study originated, the objective of the study, the demographic group under study, the size of the sample used, the research methods employed, the key concepts explored, and the principal findings that pertain to the questions posed in the scoping review. The method of synthesizing and depiction of the findings might evolve as we progress through the scoping review process

Ethics and Dissemination

Ethics review will not be required, as only publicly available data will be analyzed. Findings from the scoping review will be published in a peer-reviewed journal and disseminated to health professionals and policy-makers involved in NCD care.

DISCUSSION

The results of this scoping review will be exploring the existing evidence on various MA scales used in select NCDs such as T2DM, HTN, CAD, and BA/COPD. The review will explore in depth the psychometric characteristics of these scales, including their sensitivity, specificity, convergent validity, and reliability metrics.

The review will also identify the gaps or deficiencies in the current MA scales. This critical analysis will help us understand the limitations of the existing scales and provide insights into areas that need improvement.

In addition, the review will contribute to refining the theoretical definition of MA. It will help us understand the various domains that define MA, thereby providing a comprehensive framework for assessing medication adherence.

Finally, based on the findings of the review, we will propose potential items to be included in the new MA scale. These items will be designed to address the identified gaps in the existing scales and enhance the scale's effectiveness in assessing medication adherence in the context of the selected NCDs. This endeavor will ultimately aid in the development of a more robust and reliable MA scale.

REFERENCES

1. Burkhart PV, Sabaté E. Adherence to Long-Term Therapies: Evidence for Action. J Nurs Scholarsh. 2003;35(3):207–207.
2. Non communicable diseases [Internet]. [cited 2023 Dec 13]. Available from: https://www.who.int/news-room/fact-sheets/detail/noncommunicable-diseases
3. Currie CJ, Peyrot M, Morgan CL, Poole CD, Jenkins-Jones S, Rubin RR, et al. The impact of treatment noncompliance on mortality in people with type 2 diabetes. Diabetes Care. 2012 Jun;35(6):1279–84.
4. Sankar UV, Lipska K, Mini GK, Sarma PS, Thankappan KR. The adherence to medications in diabetic patients in rural Kerala, India. Asia Pac J Public Health. 2015 Mar;27(2):NP513-523.
5. Rao CR, Kamath VG, Shetty A, Kamath A. Treatment Compliance among Patients with Hypertension and Type 2 Diabetes Mellitus in a Coastal Population of Southern India. Int J Prev Med. 2014 Aug;5(8):992–8.
6. Balasubramanian A, Nair SS, Rakesh PS, Leelamoni K. Adherence to treatment among hypertensives of rural Kerala, India. J Fam Med Prim Care. 2018 Feb;7(1):64–9.
7. Chakma JK, Gupta S. Lifestyle and Non-Communicable Diseases: A double edged sword for future India. Indian J Community Health. 2014 Dec 31;26(4):325–32.
8. Osterberg L, Blaschke T. Adherence to Medication. N Engl J Med. 2005 Aug 4;353(5):487–97.
9. Unni EJ, Farris KB. Unintentional non-adherence and belief in medicines in older adults. Patient Educ Couns. 2011 May;83(2):265–8.
10. Thomas D, Meera NK, Binny K, Sekhar MS, Kishore G, Sasidharan S. Medication adherence and associated barriers in hypertension management in India. 2011

Jan 1;6(1):9.

11. Nguyen TMU, La Caze A, Cottrell N. What are validated self-report adherence scales really measuring?: a systematic review. Br J Clin Pharmacol. 2014 Mar;77(3):427–45.
12. Hamrahian SM, Maarouf OH, Fülöp T. A Critical Review of Medication Adherence in Hypertension: Barriers and Facilitators Clinicians Should Consider. Patient Prefer Adherence. 2022;16:2749–57.
13. Chapter 11: Scoping reviews. In: JBI Manual for Evidence Synthesis [Internet]. JBI; 2020 [cited 2023 Dec 14]. Available from: https://jbi-global-wiki.refined.site/space/MANUAL/4687342/Chapter+11%3A+Scoping+reviews
14. An Evidence-Based Approach to Scoping Reviews - Khalil - 2016 - Worldviews on Evidence-Based Nursing - Wiley Online Library [Internet]. [cited 2023 Dec 14]. Available from: https://sigmapubs.onlinelibrary.wiley.com/doi/epdf/10.1111/wvn.12144
15. Tricco AC, Lillie E, Zarin W, O'Brien KK, Colquhoun H, Levac D, et al. PRISMA Extension for Scoping Reviews (PRISMA-ScR): Checklist and Explanation. Ann Intern Med. 2018 Oct 2;169(7):467–73.
